# A factor integrating transcription and repression of surface antigen genes in African trypanosomes

**DOI:** 10.1073/pnas.2531377123

**Published:** 2026-02-03

**Authors:** María Agustina Berazategui, Ione Goodwin, Lianne I. M. Lansink, Keith Gull, Gloria Rudenko, Jack D. Sunter, Joana R. C. Faria, Richard J. Wheeler, Calvin Tiengwe

**Affiliations:** ^a^Department of Life Sciences, Imperial College London, London SW7 2AZ, United Kingdom; ^b^Department of Biology, University of York, York YO10 5DD, United Kingdom; ^c^Sir William Dunn School of Pathology, University of Oxford, Oxford OX1 3RE, United Kingdom; ^d^Department of Biological and Medical Sciences, Oxford Brookes University, Oxford OX3 0BP, United Kingdom; ^e^School of Biological Sciences, Institute for Immunology and Infection Research, University of Edinburgh, Edinburgh EH9 3FL, Scotland, United Kingdom

**Keywords:** antigenic variation, monoallelic exclusion, expression site body (ESB), transcriptional regulation, trypanosomes

## Abstract

Antigenic variation in *Trypanosoma brucei* (*T. brucei*) requires monoallelic expression of one variant surface glycoprotein (VSG) from one of the subtelomeric bloodstream form (BSF) expression sites (BESs). This transcription is unusually mediated by RNA polymerase I (RNA Pol I) and occurs in a specialized nuclear body, the expression site body (ESB). While factors promoting active BES transcription and silencing inactive BESs are known, how these opposing activities are integrated remains unknown. Here, we identify ESBX (Tb927.3.1660) as a BSF-specific ESB protein necessary for this coordination. We show that ESBX RNAi knockdown prevents RNA Pol I localizing to the ESB and reduces active BES transcription, while also derepressing inactive BESs with low processivity transcription. Conversely, ESBX overexpression weakly activates inactive BESs in a distinct manner from ESBX knockdown, leading to processive transcription, without disrupting the active BES or forming supernumerary ESBs. ESBX knockdown causes a similar transcriptomic defect to ESB1 and VEX2 knockdown combined, establishing ESBX as a key factor linking transcriptional activation of the active BES with inactive BES silencing through the VSG exclusion (VEX) phenomenon. This allows us to suggest models for understanding the establishment and maintenance of monoallelic expression critical for parasite immune evasion.

## Significance

African trypanosomes require antigenic variation to evade the host immune system. Individual trypanosomes express one variant surface glycoprotein (*VSG*) surface antigen gene from one of the largest known antigen gene families among all pathogens. The expression site body (ESB) is a dedicated subnuclear compartment central to this elegant pathogenicity mechanism. It is involved in both active *VSG* expression activation and inactive *VSG* silencing. We identify ESBX as the first protein required for both functions, giving insight into the critical balance of activation and silencing necessary for antigenic variation. This establishes a framework for understanding monoallelic expression, providing molecular insights into how pathogens regulate antigen expression to evade host immunity.

The African trypanosome represents an extreme case of a molecular “arms race” between a pathogen and its mammalian host. A single trypanosome expresses only one major antigen, the Variant Surface Glycoprotein (VSG), covering the entire cell surface in life cycle stages that infect the host or are preadapted for transmission to the host (1). This arms race has led to a genomic store house of thousands of variants of the *VSG* gene plus mechanisms for the generation of new VSG mosaics. Hence, this monoallelic VSG expression allows the parasite to maintain long term infections, evading the host adaptive immune system.

Unusually, the single active *VSG* gene is transcribed by RNA polymerase I (Pol I) (2) from a specialized bloodstream form (BSF) telomeric expression site (BES). A set of bloodstream expression site (BES)-associated genes and the single, terminal, *VSG* gene is coregulated due to polycistronic organization, with one or two promoters at the start of the unit (3–5). Although only one gene is expressed in an individual cell there are several different telomeric BESs. Switching of the *VSG* in the expressed BES is achieved by transcriptional activation of an inactive BES (4) or replacement, by recombination, of the *VSG* in the active BES with one of the ~2,500 *VSG* gene/pseudogene variants elsewhere in the genome (6, 7).

The active BES is found in a specialized Pol I-containing, extranucleolar, nuclear structure termed the expression site body (ESB) (8), from which inactive BESs are excluded (8). The ESB is present only in BSF parasites (8), despite procyclic forms (PCFs) (in tsetse fly) also selectively employing Pol I-dependent transcription of their invariant surface coat (procyclin) (9). Recent research has revealed the importance to monoallelic expression of telomere-associated factors (10–12), the inositol phosphate pathway (13, 14), epigenetic regulators (15–17), histone chaperones (18, 19), chromatin remodelers (20, 21), and SUMOylation (22, 23). In particular, the VEX proteins link an ESB-located exclusion phenomenon (24–26) to an active *VSG* gene mRNA-processing capability (27), further defining the structural elaborations associated with high-level expression of the single expressed *VSG* gene in the active BES in BSF nuclei.

Recently, greater insight to the control of monoallelic *VSG* expression was obtained by our discovery of the first ESB-specific protein (ESB1) (28). Localized only at the active BES and expressed only in mammalian infectious BSF trypanosomes, ESB1 is located near the active *VSG* Promoter, is essential for *VSG* expression and its overexpression activates inactive *VSG* promoters (28). The discovery of ESB1 gave the first insight into proteins that locate and function specifically at the ESB. Furthermore, ESB1 is required for recruitment of some, but not all, of the known ESB components (28), revealing that the ESB possesses separately assembled subdomains. VEX2 is an important component of one such subdomain and this theme was expanded by recent VEX2-proximity proteomes which identified ESB2 and ESB3, which posttranscriptionally fine-tune the levels of BES transcripts produced at the ESB (29). This emphasized the importance of VEX2 in BES transcription and RNA processing, besides its exclusion function, but ESB1 has remained the only known ESB transcription activating component.

Here, our conjecture was that ESB1 was unlikely to be the sole protein with such properties. We now report the identification of another ESB-specific protein, revealing its function and properties as a member of the ESB regulatory molecular consortium. Moreover, our comparisons of the mutant phenotypes and function of ESB1, VEX2, and this protein provide models likely to be helpful to a fuller understanding of antigenic variation in African trypanosomes.

## Results

### Approach to Identify ESB-Associated Proteins.

After considering several experimental options, we used two complementary screens for identification of ESB-associated proteins–one biochemical and one bioinformatic. First, we used ESB1 as the “bait” protein in a proximity-dependent biotinylation proteomic-based approach. Second, we used the extensive transcriptome datasets of different *Trypanosoma brucei* (*T. brucei*) life cycle stages, identifying transcripts whose abundance correlates with ESB1 transcripts. Recognizing diverse methodological caveats – such as nonspecific proximity labeling and mixed populations in transcriptional profiling – we reasoned that overlap between these two strategies would give high confidence candidates.

### Identifying ESB1-Proximal Proteins.

To identify candidate proteins proximal to ESB1, expression constructs were designed to express ESB1 fused at its N and C termini with biotin ligases miniTurbo (mT) and TurboID (30) (*SI Appendix*, Fig. S1*A*) in a cell line expressing Halo::RPA2 (Pol I second largest subunit). Hence, four *T. brucei* BSF cell lines were generated. Incubation with biotin and detection of the biotinylated product using fluorescently labeled streptavidin showed biotinylation present as a nuclear signal with a single focus per nucleus colocalized with the extranucleolar RPA2 focus, illustrated using the 6× HA::mT::ESB1 cell line which produced the strongest biotinylation signal (*SI Appendix*, Fig. S1 *B* and *C*). This matches the previously described ESB1 localization (28) and suggesting enriched biotinylation at the ESB.

We therefore performed streptavidin affinity purification of the biotinylated products from all four cell lines, including an untagged parental cell line as a control (*SI Appendix*, Fig. S1 *D* and *E*), subjected the purified material to label-free quantitative proteomics, and quantified enrichment relative to untagged parental cells for two replicates of each cell line (*SI Appendix*, Fig. S1*F*). We assessed enrichment of candidate proteins in the four different cell lines, accepting candidates which were at least Log_2_ fold change (FC) > 3 in one experiment with any of the four cell lines (Dataset S1). Additional proteins meeting enrichment thresholds were excluded following manual curation based on established subcellular localization and TriTrypDB (31) functional annotations. ESB1 was also strongly enriched and met these inclusion criteria. Given that previous work identified the ESB as a highly SUMOylated focus (22), we included Tb927.2.2460, which contains a UBC9 (SUMO E2) domain, but fell just outside the above criteria (Dataset S1).

### Identifying Transcripts with ESB1-Like Expression Profile.

As an independent, complementary approach we searched transcript abundance datasets using TriTrypDB (31) to identify transcripts with an abundance profile across life cycle stages consistent with an ESB function. While there is Pol I-dependent expression of surface coat protein-coding genes across the *T. brucei* life cycle, an ESB is present in the BSF, not present in PCFs and unlikely to be present in epimastigote or metacyclic forms. Hence, we used this pattern of expression to identify possible ESB components. We set thresholds for inclusion guided by the expression pattern of ESB1 (Tb927.10.3800). We searched the differentiation RNAseq dataset of Doleželová et al. (32) using TriTrypDB (31), selecting a minimum cut-off of 3.0-fold difference (ESB1 had a 4.4-fold difference). We also searched the Vasquez et al. (33) BSF versus PCF mRNA abundance and ribosome profiling dataset using TriTrypDB (31) using cut-offs of 2.6 and 6.0-fold difference respectively (equal to the ESB1 fold difference).

This approach identified 175 genes (Dataset S2). These were then winnowed manually by removing: i) previously characterized genes, ii) surface coat proteins (VSGs, ESAGs, ISGs), iii) metabolic enzymes and transporters, iv) cytoskeletal and motor proteins, and v) proteins having nonnuclear subcellular localizations. This left a set of 79 genes. Then, genes which had been tagged in BSF during the identification of ESB1 and found not to localize to the ESB (28, 34) were removed, leaving a set of 48 genes. A subsequent comparison of this set with the set of 26 candidates from the proximity labeling approach above showed only two overlaps: Tb927.3.1660 and Tb927.10.3800 (ESB1, the bait protein) (Datasets S1 and S2), which had distinctive expression profiles across natural and experimentally induced life cycle stage transitions (*SI Appendix*, Fig. S2 *A*–*D*) (32, 35–37).

Detailed analysis of the transcript expression data revealed that Tb927.3.1660 shows particularly strong stage-specific regulation, with ribosome occupancy data suggesting almost no translation in PCF (*SI Appendix*, Fig. S2 *E* and *F*) (33, 38). This expression pattern, similar to that of the known ESB component ESB1, further supported Tb927.3.1660 as a candidate ESB-associated protein.

### Subcellular Localization Confirms One ESB Protein Candidate.

To test whether Tb927.3.1660 was indeed a good ESB protein candidate in the context of the proximity labeling specificity and as a prelude to questioning the wider transcript abundance analysis we first asked where the 26 candidate proteins from the proximity labeling screen localized within the cell. As none were tested in our previous screen which identified ESB1 (28, 34), we determined their localization. Using N- and C-terminal tagging with mNeonGreen and native fluorescence microscopy in BSFs we successfully determined the localization of 24 out of 26.

**Fig. 1.**
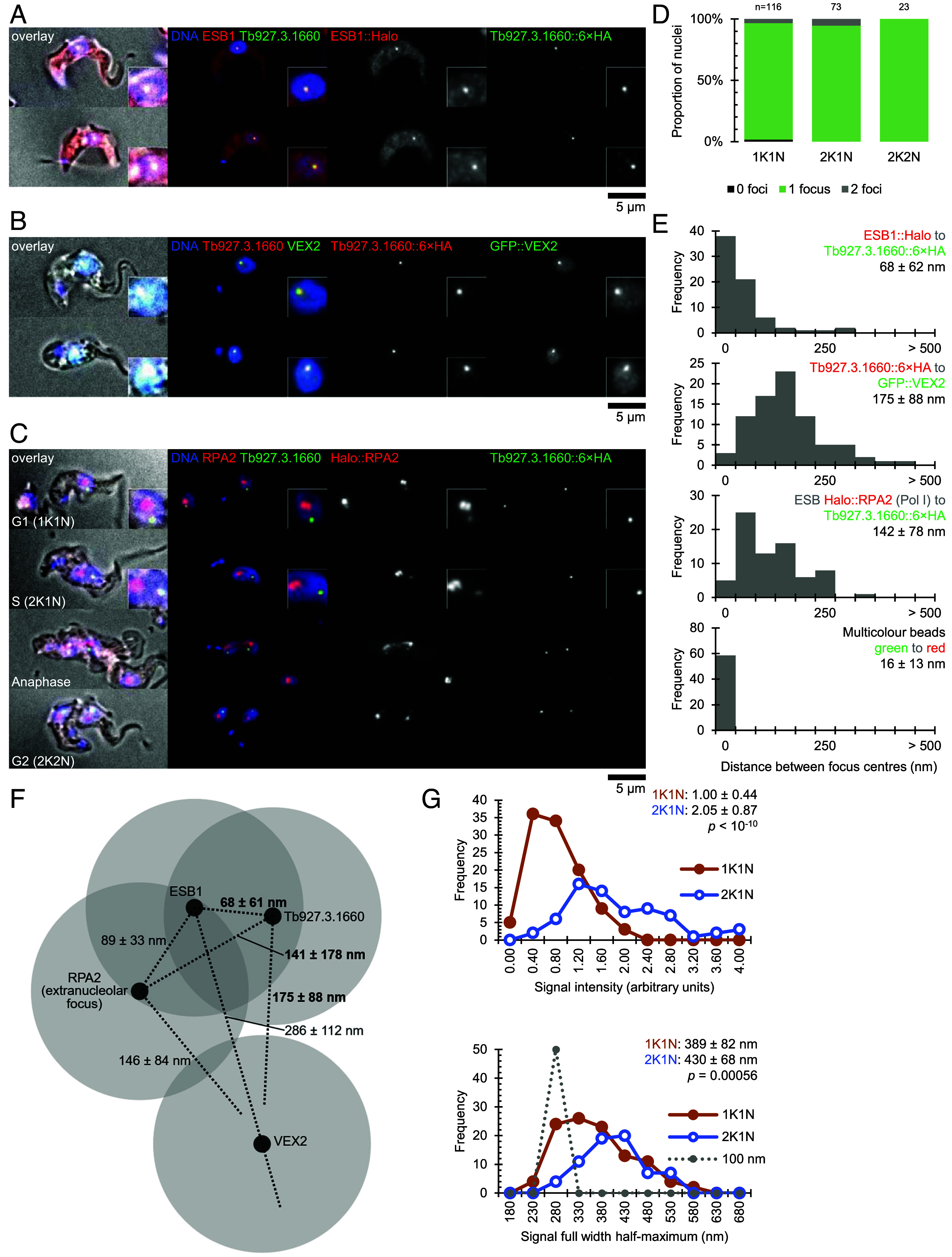
*Tb927.3.1660* encodes an ESB-specific protein ESBX positioned close to RPA2 and ESB1. (*A*) Colocalization of ESB1::Halo (red) and Tb927.3.1660::6× HA (green) in bloodstream form (BSF) trypanosomes. Representative images showing merged channels and individual channels: DNA (blue), ESB1::Halo, and Tb927.3.1660::6× HA. *Insets* show magnified views of the nuclear region. (*B*) Colocalization of Tb927.3.1660::6× HA (red) and GFP::VEX2 (green) in BSF trypanosomes. Representative images showing merged channels and individual channels: DNA (blue), Tb927.3.1660::6× HA and GFP::VEX2. *Insets* show magnified views of the nuclear region. (*C*) Colocalization of RPA2::Halo (red) and Tb927.3.1660::6× HA (green). Representative images of G1, anaphase, and telophase cells showing merged channels and individual channels: DNA (blue), RPA2::Halo, and Tb927.3.1660::6 × HA. *Insets* show magnified views of the nuclear region. (*D*) Quantification of Tb927.3.1660::6× HA nuclear focus number across different cell cycle stages (1K1N, 2K1N, 2K2N). Graph shows the proportion of nuclei containing 0 (black), 1 (green), or 2 (gray) foci. Numbers above bars indicate total nuclei analyzed (*n*) for each cell cycle stage. (*E*) Histogram showing distance measurements between signal focus centers in 1K1N cells: Halo::ESB1 and ESBX::6× HA, ESB Halo::RPA2 extranucleolar focus and ESBX::6× HA, ESBX::6× HA and GFP::VEX2 and, finally, red and green fluorescence images of 100 nm wide multicolor fluorescent beads. (*F*) Diagram summarizing foci center point (black circle) separations and approximate size (gray circles, based on ESBX focus width). Lines lengths are drawn to scale, proportional to measured mean separation for a particular protein pair. Measurements either, bold, from (*E*) or, regular, from ref. 28. (*G*) Measurement of heterozygous ESBX::6× HA signal intensity and focus width in 1K1N and 2K1N cells. Histograms showing distribution of signal intensity (normalized to 1K1N mean) or full width half-maximum (FWHM) measurement. FWHM also plotted for nominally 100 nm-wide fluorescent beads, with a predicted FWHM of 282 nm (Airy disk FWHM 182 nm plus 100 nm width). *P* values from two-tailed unpaired *t* test.

Different localizations emerged from this survey but 19 of 24 were nuclear (Dataset S1). However, very significantly, only the product of Tb927.3.1660 localized to a single focus per nucleus when tagged at either the N or C terminus with mNeonGreen. Moreover, the Tb927.3.1660::6× HA focus was near-coincident with the ESB, as determined by localization of ESB1 in a cell line also expressing ESB1::Halo (Fig. 1*A*), and a cell line also expressing GFP::VEX2 (Fig. 1*B*). We also asked whether Tb927.3.1660::6× HA localized to the ESB as defined by the presence of Halo-tagged RNA Pol I protein subunit, RPA2, which was the case (Fig. 1*C*). Furthermore, Tb927.3.1660::6× HA localized to a single focus in G1 cells (1 kinetoplast and 1 nucleus, 1K1N) and G2 cells (2K1N); two foci appearing only in nuclei in mitotic anaphase, each focus being in each segregating half spindle, and a single focus in each nucleus of postmitotic cells (2K2N) (Fig. 1 *C* and *D*). As described above, based on transcriptomic databases, the Tb927.3.1660 protein is not expressed in PCFs. When endogenously tagged with a fluorescent protein in PCFs only background cytoplasmic fluorescence was observed (39). This pattern fits perfectly with the known life cycle, spatial and cell cycle pattern of the ESB as defined by RPA2 and ESB1 proteins previously described (8, 28). We therefore focused our attention on this protein, considering that it fitted the criteria for an ESB-specific protein and we named it ESBX (where X indicates cross-connectivity, which we expand on below).

### ESBX Localizes Close to Pol I and ESB1 within the ESB.

The ESBX focus had near perfect colocalization with the RPA2 and ESB1 markers of the ESB. To determine the precise spatial correspondence to this transcription-associated ESB machinery we measured the distance between focus centers. ESBX to ESB1 distance was 68 ± 62 nm (Fig. 1 *E* and *F*), comparable to the ESB1 to ESB RPA2 distance we previously observed (28). ESBX to ESB RPA2 distance was 142 ± 78 nm (Fig. 1 *E* and *F*), comparable to the VEX2 to ESB RPA2 distance we previously observed (28). ESBX to VEX2 distance was a little larger, at 175 ± 88 nm (Fig. 1 *E* and *F*). Measurement precision was confirmed to be high using multicolor fluorescent beads (Fig. 1*E*) and allows us to present a map of the centers of each subcompartment of the ESB (Fig. 1*F*). This localization is consistent with ESBX having been identified by proximity labeling using ESB1 and being a bona fide component of the ESB.

To investigate the duplication of the active BES within the ESB during S phase and segregation to daughter nuclei at mitosis, we first generated cell lines with either one or both ESBX alleles tagged with 6× HA. Both heterozygous and homozygous tagged lines showed normal growth over 96 h, allowing us to confidently analyze ESBX dynamics throughout the cell cycle. Heterozygous ESBX::6× HA remained in a singular focus in cells with a single nucleus, but the focus increased in signal intensity and was wider in cells after nuclear genome duplication, in 2K1N cells compared to 1K1N cells. The focus was twice as bright, but only slightly wider (Fig. 1*G*)–consistent with a doubling of ESBX content and doubling of volume with a correspondingly smaller increase in width. By comparison to reference fluorescent beads, we infer an approximate G1 width of the ESBX compartment of ~200 nm.

### ESBX Has SUMO-Interacting and BRCA Domain-Containing Features.

To gain insight into potential functions, we analyzed the predicted structure and evolutionary conservation of ESBX. The predicted structure reveals a small, structured N and larger structured C terminal domain, connected by a >300 amino acid unstructured region (*SI Appendix*, Fig. S3*A*). Recent SUMOylation interaction motif (SIM) prediction tools [GPS-SUMO 2.0 (40)] predict a SIM. The N-terminal domain has a predicted Rossman fold, and the C terminal domain has two predicted BRCA C terminal (BRCT) domains. This has similarities to the ESB1 predicted structure, which has a comparable large central unstructured region, with predicted SIMs and a C terminal BRCT domain but which displays a dissimilar small N-terminal domain (a RING U-box domain) (41, 42). Diverse kinetoplastids have orthologs of ESBX (*SI Appendix*, Fig. S3*B*) with a conserved domain architecture (*SI Appendix*, Fig. S3*C*) further indicating lineage-specific adaptation of existing machinery to develop the ESB.

### ESBX Depletion Causes Growth Arrest and Cell Cycle Defects.

**Fig. 2.**
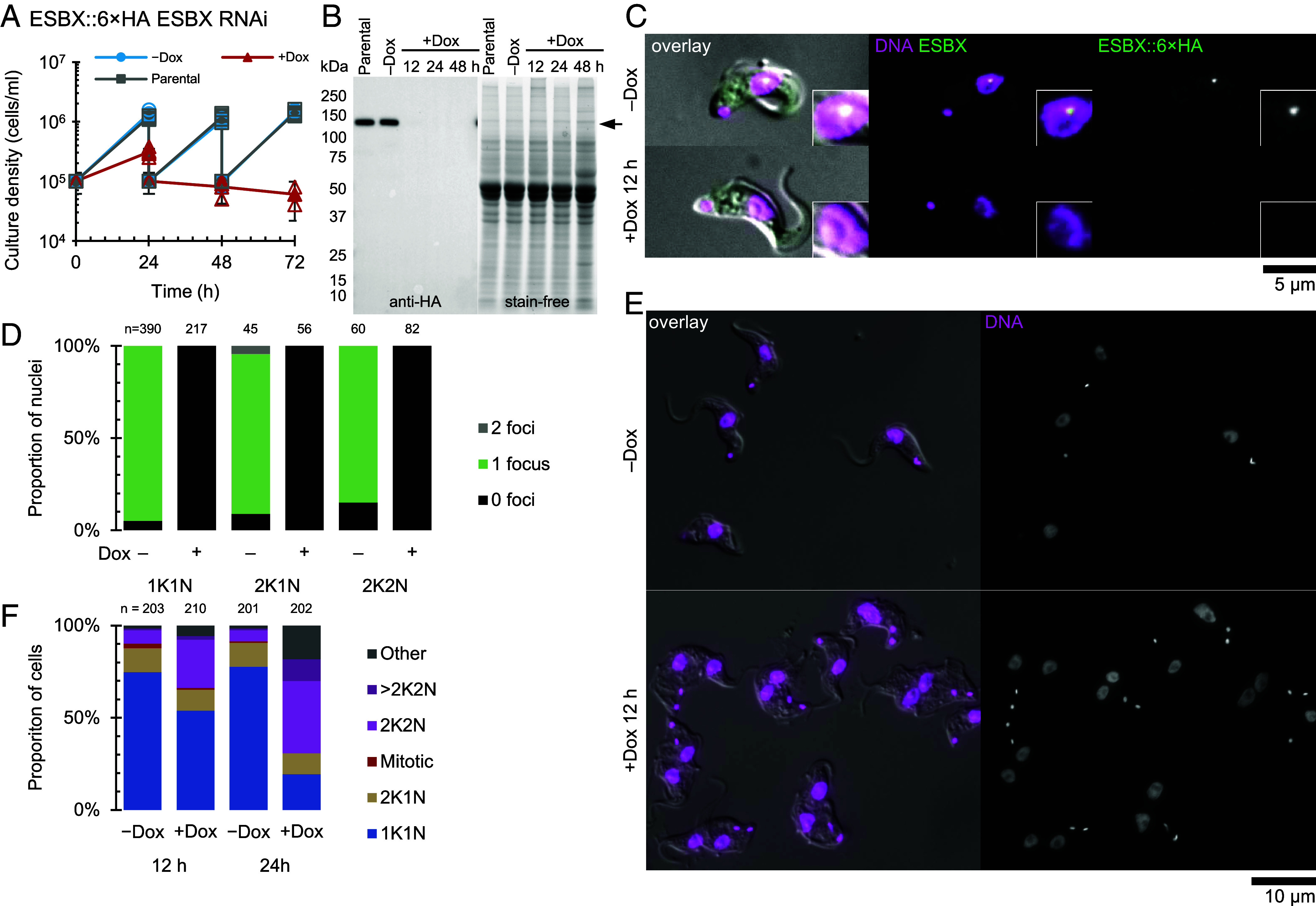
ESBX depletion causes growth arrest and cell cycle defects. (*A*) Growth of parental (ESBX::6× HA) and ESBX RNAi-inducible cells (ESBX::6× HA ESBX RNAi) treated with (+Dox, 1 μg mL^−1^) or without (−Dox) doxycycline, maintained with 24 hourly subculture to 1 × 10^5^ cells mL^−1^. Data points indicate individual replicates (*n* = 4 technical replicates of a single representative clone out of three independent clones). (*B*) Western blot analysis of ESBX::6× HA protein levels in parental and ESBX RNAi-inducible cells without (−Dox) and 12, 24, and 48 h after Doxycycline addition (+Dox). Whole cell lysates were probed with anti-HA antibody followed by HRP-conjugated secondary antibody and detected by chemiluminescence or stain-free imaging. Representative blot of one clone is shown. Molecular weights (kDa) are shown on the *Left*. The arrowhead indicates ESBX::6× HA. (*C*) Localization of ESBX::6× HA without (−Dox) and 12 h after doxycycline addition (+Dox). Representative images of 1K1N cells immunostained with anti-HA antibody (ESBX::6× HA, green) and DAPI (DNA). Images show merged and individual channels with insets showing magnified views of nuclear regions. (*D*) Number of ESBX::6× HA foci per nucleus across cell cycle stages (1K1N, 2K1N, 2K2N) without (−) and 12 h after (+) doxycycline treatment. Numbers above bars indicate total nuclei analyzed (*n*) for each category. (*E*) Cell morphology and DNA content analysis of ESBX RNAi cells. Representative images of ESBX::6× HA/ESBX RNAi treated without (−Dox) and with (+Dox, 12 h) doxycycline. Phase contrast images and DNA stained with DAPI (magenta) are shown as separate and merged channels. (*F*) Quantification of cell cycle stages in ESBX RNAi cells 12 or 24 h without (−Dox) or with (+Dox) doxycycline. Cell populations were categorized into 1K1N (one kinetoplast, one nucleus), 2K1N (two kinetoplasts, one nucleus), mitotic, 2K2N (two kinetoplasts, two nuclei), >2K2N (more than two kinetoplasts, two nuclei) and other. Data are shown for three independent clones, numbers above bars indicate total nuclei analyzed for each condition.

To determine ESBX function, we generated a BSF cell line expressing ESBX::6× HA with a genome-integrated inducible ESBX RNAi construct. Three independent clones were analyzed, and representative data for one clone are presented in Fig. 2. Induction of RNAi knockdown by doxycycline addition had an early, dramatic effect such that by 24 h cell growth had effectively ceased (Fig. 2*A*), likely indicative of rapid ESBX turnover. Western blot analysis using anti-HA antibodies showed only trace amounts of ESBX::6× HA at 12 h and none by 24- and 48-h postinduction (Fig. 2*B*), again a result that would fit with rapid ESBX turnover. Immunofluorescence microscopy revealed that nearly all cells (>90%) had lost their ESBX::6× HA nuclear focus by 12 h, compared to only 5% of cells without detectable foci before induction (Fig. 2 *C* and *D*).

ESBX depletion produced a profound effect on cell cycle progression with significant accumulation of postmitotic 2K2N cells (Fig. 2*E*), initially (12 h) showing an increase from 6 to 29%, while 1K1N cells decreased from 77 to 51% (Fig. 2*F*) reminiscent of the phenotype observed upon VSG depletion (43). At 24 h, 1K1N cells further decreased to 25% while 2K2N increased to 37%, and 25% of cells displayed abnormal configurations (>2N and/or >2K) (Fig. 2*F*).

### ESBX Is Necessary for Active BES Expression and Inactive BES Exclusion.

**Fig. 3.**
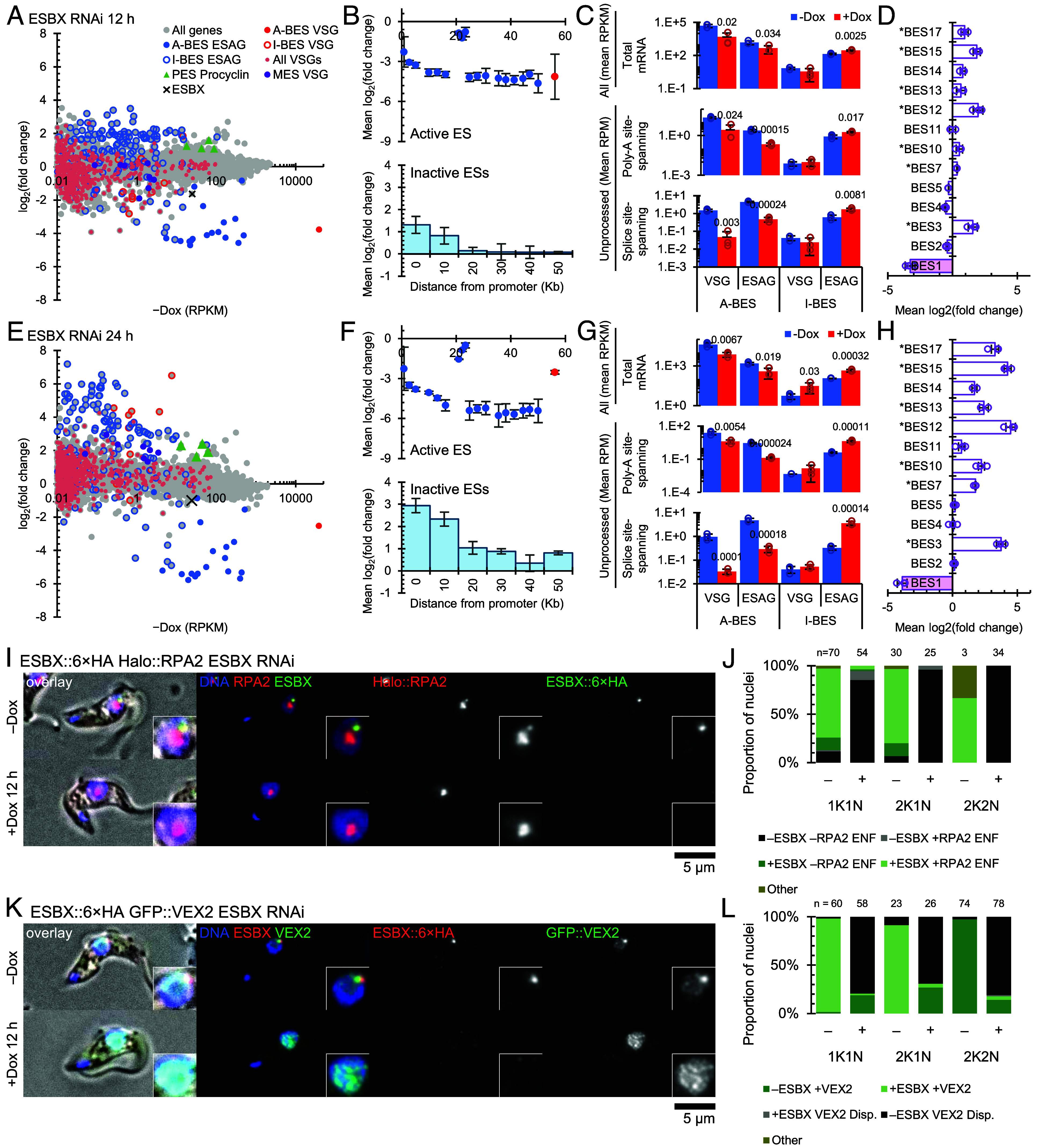
ESBX is necessary for active BES expression and inactive BES repression. (*A*) Plots of mean fold-change at 12 h after ESBX RNAi induction relative to uninduced cell line plotted against mean transcript abundance (reads per kilobase per million uniquely mapped reads, RPKM) of the uninduced cell line. Each data point represents mean of three samples for a single transcript. Color-coding identifies: active BES *VSG* (A-BES VSG), active BES *ESAGs* (A-BES ESAG), inactive BES *VSGs* (I-BES VSG), inactive BES *ESAGs* (I-BES ESAG), all *VSGs*, procyclin (PES Procyclin), and metacyclic *VSGs* (MES *VSG*). (*B*) Mean fold change relative to uninduced cell line of each *VSG* and *ESAG* gene in the (*Top*) active and (*Bottom*) inactive expression sites, plotted (*Top*) by distance from the promoter or (*Bottom*) as a histogram by distance from promoter. Data points or bars represent the mean, error bars represent SD. (*C*) Mean total and unprocessed mRNA at 12 h grouped into *VSG(s)* and *ESAGs* for active (A-BES) and inactive (I-BES) BESs, showing the uninduced (−Dox) and induced (+Dox) conditions. *Top* panels show total mRNA (All), middle panels show polyadenylation site-spanning reads, *Bottom* panels show splice site-spanning reads. Bars represent the mean, error bars represent the SD of replicates, open circles represent the mean for each replicate. *P* values shown from two-tailed *t* test comparing induced to uninduced where *P* < 0.05. (*D*) Mean log2(fold change) relative to uninduced cell line across all genes in each BES at 12 h. Bars represent the mean, error bars represent the SD of the replicates, open circles represent the mean for each replicate. BES1 is the active BES, asterisks indicate that BES has two promoters. (*E*–*H*) As in (*A*–*D*) but at 24 h. In (*E*), color-coding as in (*A*). In (*G*), panel layout and statistics as in (*C*). (*I*) Colocalization of RPA2::Halo (red) and ESBX::6× HA (green) without (−Dox) and 12 h after (+Dox) doxycycline-induced ESBX knockdown. Representative images of 1K1N cells showing merged and individual channels: DNA (blue), RPA2::Halo, and ESBX::6× HA. (*J*) Number of ESBX::6× HA foci and RPA2 extranucleolar foci (ENF) per nucleus across cell cycle stages without (−Dox) and 12 h after (+Dox) doxycycline addition. Color-coded categories represent different combinations of ESBX foci and RPA2 ENF presence (+) or absence (−). Numbers above bars indicate number of cells analyzed (n) per category. (*K*) Colocalization of GFP::VEX2 (green) and ESBX::6× HA (red) without (−Dox) and 12 h after (+Dox) doxycycline-induced ESBX knockdown. Representative images of 1K1N cells showing merged and individual channels: DNA (blue), GFP::VEX2, and ESBX::6× HA. (*L*) Number of ESBX::6× HA foci and GFP::VEX2 distribution (ENF) per nucleus across cell cycle stages without (−Dox) and 12 h after (+Dox) doxycycline addition. Color-coded categories represent different combinations of ESBX foci and VEX2 foci presence (+) or absence (−) and, for VEX2, whether the signal was dispersed. Numbers above bars indicate number of cells analyzed (*n*) per category.

To test if ESBX is necessary for monoallelic expression of the single *VSG* and the *ESAGs* of the active BES, we sequenced the transcriptome of the three independent clones of the ESBX RNAi cell line before and 12 and 24 h after doxycycline induction of the knockdown (Fig. 3 *A*–*H* and *SI Appendix*, Fig. S4 *A* and *B*). This showed *ESBX* mRNA decreased threefold (RPKM 24.91 ± 0.43 to 8.19 ± 2.24, *P* = 0.0016 two-tailed *t* test, *p* = 5.640 × 10^−15^ using EdgeR) by 12 h. As *ESAG*s within BESs often have highly similar sequences, we analyzed only transcriptome sequencing reads which aligned uniquely to a predicted transcript. This ensures that we can detect changes to specific BESs, but at the cost of absolute transcript abundance information.

There was a strong rapid effect of ESBX depletion on transcript profile consistent among the three independent clonal cell lines with many significant changes at 12 h and becoming more severe by 24 h (Fig. 3 *A*–*H*, *SI Appendix*, Fig. S4 *A* and *B*, and Dataset S3). Active BES *VSG* and *ESAG* transcripts decreased dramatically in abundance (Fig. 3 *A* and *E*). Downregulation of the active BES was associated with activation of inactive BESs. The active BES transcripts decreased a little from 12 h to 24 h, while inactive BES transcripts, particularly *ESAG* transcripts, increased in abundance from 12 to 24 h postinduction (Fig. 3 *A* and *E*). We analyzed uniquely aligned reads, which confounds statements about absolute abundance of transcripts, but it is likely that the *VSG* transcripts being produced from inactive BESs is not sufficient to replace the drop of *VSG* transcripts from the active BES, hence the 2K2N cell cycle block. Procyclins, both EP and GPEET, were significantly increased from 12 h. Metacyclic *VSGs* were significantly increased at 24 h (Fig. 3 *A* and *E* and *SI Appendix*, Fig. S4 *A* and *B*). While ESBX was identified by proximity and expression profile similar to ESB1, its phenotype is unlike ESB1 conditional knockout or RNAi knockdown. There, transcripts from both active and inactive BESs decreased (while Procyclins and metacyclic *VSGs* increased a little) (28) (*SI Appendix*, Fig. S5).

Looking at the active BES, transcript abundance decreased more for genes positioned further from the promoter at both 12 and 24 h postinduction (Fig. 3 *B* and *F*, *Top*). This suggests ESBX is necessary for high processivity of active BES transcription and that in its absence less processive ESBX-independent transcription still occurs. This is similar to ESB1 cKO and RNAi (28). Change in transcript abundance from inactive BESs did not have a clear correlation with distance from the promoter, although promoter-proximal *ESAGs* tended to increase more than promoter-distal genes (Fig. 3 *B* and *F*, *Bottom*), suggesting somewhat inefficient transcript elongation comparable, somewhat intriguingly, to the phenotype of ESB1 overexpression (28).

Transcript abundance is the result of the balance of transcript production and degradation. To analyze the state of transcription and transcript processing from the active and inactive BESs, we asked if there were changes to unprocessed transcripts using the number of reads spanning polyadenylation or spliced leader acceptor sites (Fig. 3 *C* and *G* and Dataset S3). Decreased active BES *VSG* and *ESAG* transcript abundance had an associated decrease in unprocessed transcript at both 12 and 24 h postinduction (Fig. 3 *A* and *E*). This is either due to reduced transcription or increased transcript processing – the former is much more likely given the drop in overall active BES *VSG* and *ESAG* transcript abundance. This is again similar to ESB1 cKO (28).

Increased “inactive” BES *VSG* and *ESAG* transcript abundance had an associated increase in unprocessed *ESAG* but not *VSG* transcript (Fig. 3 *A* and *E*). Increased unprocessed transcript indicates some combination of increased transcription and/or inefficient/insufficient transcript processing. This is unlike ESB1 cKO where inactive BES transcript abundance decreased, and unprocessed transcript decreased in correlation with that, indicating decreased transcription (28).

Last, we asked which BESs tended to activate. Change in transcript abundance varied between BESs and at both 12 and 24 h postinduction, transcripts from BES3, 12 and 15 were most increased (Fig. 3 *D* and *H*). There was a perfect correlation with increased BES activity and number of promoters, at 24 h the seven BESs with two Pol I promoters are top seven in fold increase. The highest single promoter BES, BES14, contains the same *VSG* as the two promoter-containing BES12 (4), and imperfect unique read mapping may be responsible for the apparent high level of BES14 expression. This pattern is similar to ESB1 overexpression (28) and very similar to the VEX2 RNAi phenotype (25) (*SI Appendix*, Fig. S5).

This sequencing analysis is from cell populations, so we cannot directly determine if each cell is tending to activate transcription from multiple BESs or if cells are tending to switch to a different active BES. Additionally, some cells may be expressing procyclin and metacyclic VSG in addition to or instead of BSF VSG. However, VEX2 RNAi was shown to be a multiexpression situation with transcription from multiple BESs, preferring two promoter BESs, at the single cell level, (25, 26) so the high similarity of the ESBX and VEX2 RNAi population RNAseq phenotype strongly suggests multiexpression.

### The ESB Structure Is ESBX-Dependent.

Reduction of active BES transcription upon ESBX RNAi knockdown suggests that RNA Pol I may be lost from the ESB and/or that the ESB disassembles. We tested this by using the ESBX::6× HA-expressing ESBX RNAi cell line, inducing ESBX knockdown, and observing the localization of HaloTagged-RPA2 (Fig. 3 *I* and *J*). Before RNAi induction, ESBX foci were positioned with the extranucleolar RPA2 focus, as expected (Fig. 1*C* cf. Fig. 3 *I* and *J*), with approximately 90% of 1K1N cells showing colocalization of the two foci. The RPA2 extranucleolar ESB focus was lost upon ESBX knockdown (Fig. 3 *I* and *J*), with the percentage of cells with such foci dramatically decreasing from ~90% before induction to only ~10% after 12 h, indicating that ESBX knockdown effect on the active BES transcription involves disassembly of RNA Pol I from its extranucleolar ESB focus.

Analogously, the VEX2 RNAi-like effect on inactive ESs suggests that VEX2 would also be lost from the ESB upon ESBX knockdown. We tested this by endogenously tagging VEX2 with GFP in the ESBX::6× HA-expressing ESBX RNAi cell line, inducing ESBX knockdown, and observing the localization of VEX2 (Fig. 3 *K* and *L*). Before RNAi induction, VEX2 localized to a single focus per nucleus with the ESBX focus in almost all cells, as expected (Fig. 1*B* cf. Fig. 3 *K* and *L*). VEX2 localization tended to change upon knockdown, redistributing to multiple foci, often perinucleolar, along with the loss of ESBX foci (Fig. 3 *K* and *L*). As loss of VEX2 from an ESB focus phenocopies the effect on inactive BES transcript abundance from complete loss of VEX2, this indicates that the *VSG* exclusion (VEX) function of VEX2 requires its focal localization to the ESB.

### Inducible Exogenous Expression of ESBX Causes Aberrant Inactive BES Transcription.

**Fig. 4.**
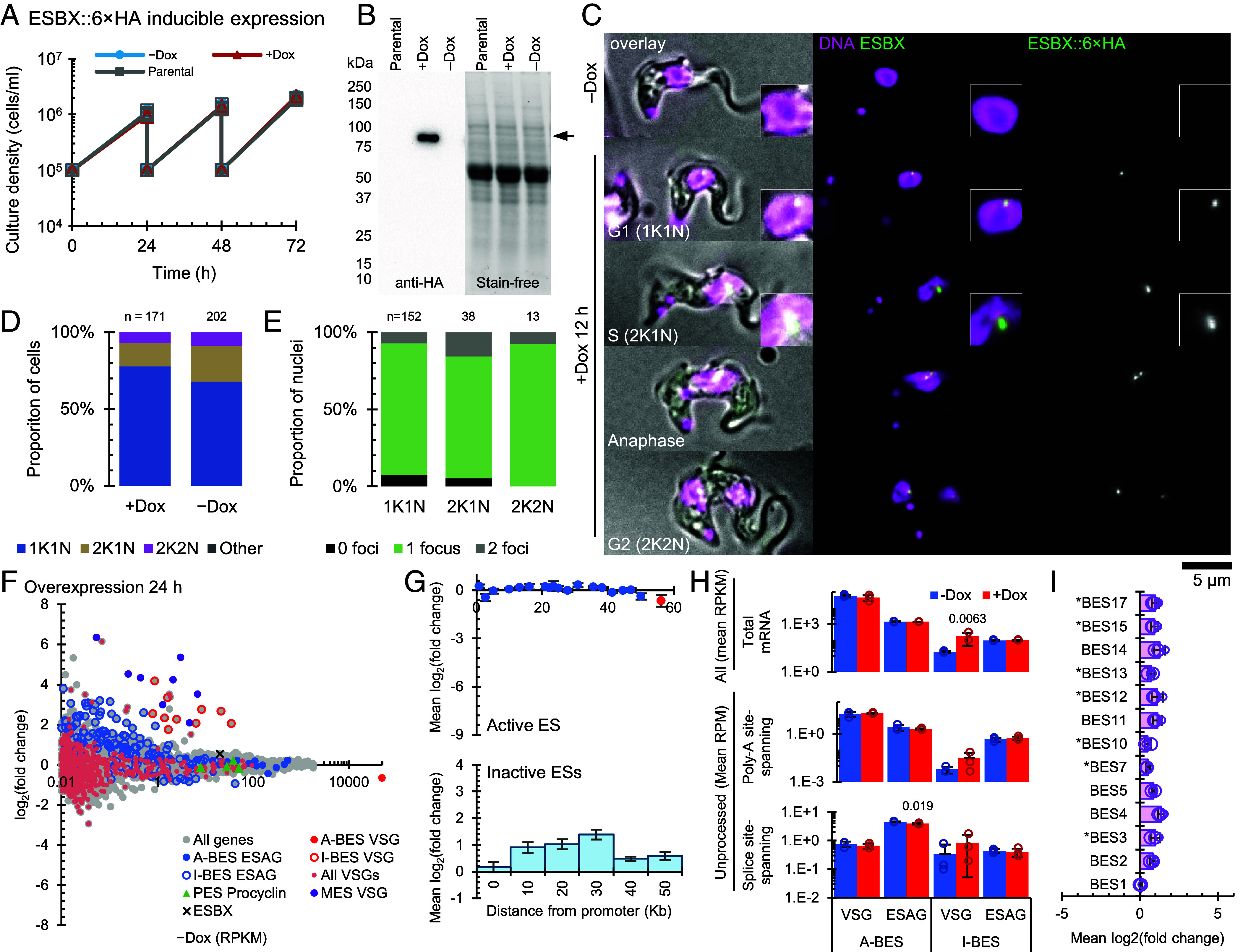
ESBX overexpression is tolerated and activates inactive BESs without forming supernumerary ESBs. (*A*) Growth of parental (ESBX::6× HA) and ESBX::6× HA inducible expression cells treated with (+Dox, 1 μg mL^−1^) or without (−Dox) doxycycline. Cells were seeded at 1 × 10^5^ cells mL^−1^ and diluted to this density every 24 h. Cell density was determined by counting at 24 h intervals. Data are means ± SD (*n* = 3 technical replicates of a single representative clone out of 3). (*B*) Western blot analysis of ESBX::6× HA protein levels in parental and ESBX inducible expression cells with and without doxycycline treatment. Cell lysates were probed with anti-HA antibody followed by HRP-conjugated secondary antibody and detected by chemiluminescence or stain-free imaging. Representative blot of one clone out of three independent experiments. Molecular weights (kDa) are shown on the *Left*. The arrowhead indicates ESBX::6× HA. (*C*) Subcellular localization of inducible ESBX::6× HA expression. Representative images of parental 2T1 and ESBX::6× HA inducible expression cells without (−Dox) and with (+Dox) doxycycline treatment (*n* = 3 biological replicates). Merged and individual channels show DNA (Hoechst, magenta) and ESBX::6 × HA (green) detected with anti-HA antibody. Images are shown for different cell cycle stages (G1 1K1N, G2 2K1N, and telophase). (*D*) Distribution of ESBX::6× HA protein without (−Dox) or with (+Dox) doxycycline treatment across the cell cycle. Percentages of cells in 1K1N, 2K1N, and 2K2N configurations are shown. (*E*) Quantification of ESBX::6 × HA nuclear foci across different cell cycle stages (1K1N, 2K1N, 2K2N) in doxycycline -treated cells. No foci were observed in untreated (control) cells. Graph shows the proportion of nuclei containing 0 (black), 1 (green), 2 (purple), or >2 (dark purple) foci. Numbers above bars indicate total nuclei counted (*n*) for each cell cycle stage in a single clone. (*F*) Plots of mean fold-change at 12 h (*A*) and 24 h (*E*) after ESBX exogenous expression induction relative to uninduced cell line plotted against mean transcript abundance (reads per kilobase per million uniquely mapped reads, RPKM) of the uninduced cell line. Each data point represents mean of three samples for a single transcript. (*G*) Mean fold change relative to uninduced cell line of each *VSG* and *ESAG* gene in the (*Top*) active and (*Bottom*) inactive expression sites, plotted by distance from the promoter. Data points represent the mean, error bars represent SD. (*H*) Mean total and unprocessed mRNA grouped into *VSG(s)* and *ESAGs* for active (A-BES) and inactive (I-BES) BESs, showing the uninduced (−Dox) and induced (+Dox) conditions. *Top* panel shows total mRNA (All), middle panel shows polyadenylation site-spanning reads, *Bottom* panel shows splice site-spanning reads. Bars represent the mean, error bars represent the SD of the replicates, open circles represent the mean for each replicate. *P* values shown from two-tailed *t* test comparing induced to uninduced where *P* < 0.05. (*I*) Mean log2(fold change) relative to uninduced cell line across all genes in each BES. Bars represent the mean, error bars represent the SD of the replicates, open circles represent the mean for each replicate. BES1 is the active BES, asterisks indicate that BES has two promoters.

We then asked how inducible expression of ESBX influenced gene expression from both the active BES and inactive BESs and whether there was any supernumerary ESB formation. Expression was achieved using a cell line with an inducible ESBX::6× HA locus in addition to the two unmodified endogenous ESBX loci. We generated clonal cell lines, noting a little variability, and selecting one with good exogenous expression, from which data is presented in Fig. 4.

Enhanced expression showed no discernible effect on growth in comparison to the uninduced sample (Fig. 4*A*). This tolerance of increased ESBX levels contrasts with the severe growth arrest following ESBX depletion, suggesting fundamentally different consequences of ESBX loss versus excess. Western blot analysis confirmed effective inducible exogenous expression (Fig. 4*B*). Without induction, ESBX::6× HA was undetectable by immunofluorescence (Fig. 4*C*), and induction caused no significant change to the proportion of cells in each cell cycle stage (Fig. 4*D*). The localization of exogenously expressed protein had no clear difference to ESBX::6× HA expressed from the endogenous locus (Fig. 1*A*), with approximately 90% of 1K1N containing one focus per nucleus (Fig. 4*E*) – it did not cause supernumerary foci.

To test the effect of excess ESBX on transcripts from active and inactive BES, we sequenced the transcriptome of the ESBX cell line containing the exogenous copy before and 24 h after doxycycline induction (Fig. 4 *F*–*I*, *SI Appendix*, Fig. S4*C*, and Dataset S3), using the same analysis of only uniquely aligned reads as for analyzing the RNAi knockdown. This showed total *ESBX* mRNA was only modestly upregulated (up 1.5-fold, RPKM 20.37 ± 1.07 to 29.82 ± 0.84, *P* = 0.00038 two-tailed *t* test, *P* = 0.0035 by EdgeR) by 24 h.

There was a strong effect of increased exogenous ESBX::6× HA expression on transcript profile, with many significant changes by 24 h. Inactive BES *VSG* and *ESAG* transcripts increased in abundance markedly, while active BES *VSG* and *ESAG* transcripts were unchanged (Fig. 4*F* and *SI Appendix*, Fig. S4*C*). Procyclin, both EP and GPEET, transcripts were unchanged, while metacyclic *VSG* transcripts were significantly increased (Fig. 4*F* and *SI Appendix*, Fig. S4*C*).

Unlike the ESBX RNAi knockdown, the effect on inactive BES *VSG* transcripts was typically more pronounced than for inactive BES *ESAGs*. We analyzed this further by looking at the difference in correlation of change in abundance of transcripts from the active and inactive BESs with distance from the BES promoter. From inactive BESs, *VSG* transcripts (found at the distal end of the BES) and *ESAG* transcripts more distal from the promoter tended to be more upregulated (Fig. 4*G*). This suggests a move to a transcriptional/processing pattern more akin to a normal active state, unlike following ESBX RNAi knockdown (Fig. 3 *B* and *F*). Both of these phenomena are unlike ESB1: ESB1 overexpression caused a bigger increase in transcript abundance for inactive BES *ESAGs* rather than *VSGs*, and promoter proximal genes tended to have more transcript increase (28), although ESBX overexpression was weak.

Analysis of unprocessed transcript did not show any large change, for both transcripts from the active and inactive expression site (Fig. 4*H* and Dataset S3). This suggests that the transcript from the inactive BESs activated by ESBX overexpression is processed with normal efficiency, unlike for inactive BESs activated by RNAi knockdown of ESBX (Fig. 3 *C* and *G*). We asked which BESs tended to be activated upon ESBX overexpression, and all BESs were broadly evenly activated, with a weak preference for activating BES4 (Fig. 4*I*). This is a different pattern to ESBX RNAi (Fig. 3 *D* and *H*), VEX2 RNAi (25, 26) or ESB1 overexpression (28) (*SI Appendix*, Fig. S5). The high quantity of inactive BES *VSG* relative to inactive BES *ESAG* transcript upon ESBX overexpression is reminiscent of the high ratio of active BES *VSG* relative to active BES *ESAG* transcript, although the quantity of the most abundant *VSG* transcript from an inactive BES was <0.5% of the active BES *VSG* transcript.

## Discussion

Over two decades after the identification of the ESB, the mechanisms underpinning monoallelic *VSG* expression in *T. brucei* are becoming clearer. The ESB operates within a network of nuclear bodies (44), in proximity to RNA processing machinery and the VEX complex (24–26). Yet, among this machinery, only ESB1 was identified as specifically promoting BES transcription.

Here, we identify ESBX as a BSF-specific ESB component. Although this protein had been previously noted among transcripts responsive to DNA double-strand breaks that trigger *VSG* switching (45) and in analyses of low-abundance mRNAs (46), its functional significance remained unexplored. Our work now demonstrates that ESBX is ESB1-proximal and essential for both active BES transcription and Pol I localization at the extranucleolar focus. ESBX is the second protein identified, after ESB1, for this transcriptional activation.

However, unlike ESB1, ESBX is also required for inactive BESs to remain in their low transcription state and ESBX depletion increases inactive BES transcription. In contrast ESB1 is essential for all BES transcription promoters (28). We only achieved a modest ESBX overexpression, likely because of rapid turnover of ESBX, which also influenced transcription, perhaps causing switching (but, if so, only in a small proportion of cells) or alternatively low-level expression from multiple BESs in all cells. Therefore, we suggest that ESBX is a key factor integrating both active BES transcription and inactive BES repression. Our current view is that ESBX is likely upstream of ESB1 in a regulatory or dependency relationship, although this may not be a strict hierarchical relationship as we can envisage a process whereby cooperative recruitment of ESBX occurs and so enhances ESB1 mediated transcription in a positively incremental manner.

ESBX being necessary to keep inactive BESs in their very low transcription state is the third identified protein factor for this VEX behavior after VEX1 and VEX2 (24, 25). ESBX RNAi knockdown causes loss of the VEX2 focus at the ESB, loss of the ESB Pol I focus itself, and activation of inactive BES, albeit to a lesser extent than VEX2 depletion (*SI Appendix*, Fig. S5). It is possible that the disruption of allelic exclusion observed following ESBX knockdown may be indirect through loss of VEX2 compartmentalization at the ESB, ESBX is likely also upstream of VEX2 in some way. However, ESBX function is distinct from VEX1 and VEX2 as they are not required for normal active BES transcription (24–26). ESBX therefore sits at the interface between BES promoter transcription activation and repression mechanisms, and a simple mechanism would be for it to enable VEX1/VEX2 in addition to ESB1 activity. The VEX mechanism is described as a sequestration-based mechanism of allelic exclusion (24–26), which builds on the physical separation of the inactive BESs from the singularity of the ESB, with its associated promoter-activating machinery (8). ESBX depletion causes both loss of the ESB Pol I focus, implying loss of the ESB, and activation of inactive BES. The mechanism of ESBX VEX function is therefore plausible by establishing the physical separation of the active BES promoter-activating machinery from the inactive BESs.

ESBX contributions may be structural, given its lack of predicted enzymatic domains, perhaps constructing a specific promoter-proximal ESB microenvironment or helping maintain integrity of the conglomerate of associated nuclear bodies (44) that make up the ESB. The combination of protein–protein interaction-associated BRCT domains with intrinsically disordered regions is consistent with the formation of a condensate by a phase separation mechanism of membrane-less compartment formation (47), for example BRCT domain-containing proteins involved in DNA damage responses (48), suggesting this as a testable hypothesis for future work.

ESBX fulfills earlier predictions that the ESB would contain both specialized RNA processing factors distinct from those required for rRNA transcription and essential protein components required for ESB assembly and maintenance of singularity (49). Neither ESB1 nor ESBX has a high proportion of positively charged amino acids characteristic of nucleolar targeting (50, 51), perhaps explaining nucleolar exclusion to assist targeting to the ESB. VEX2, important for ESB integrity (25), may also contribute: VEX2 is a putative RNA or RNA:DNA helicase, orthologs of which are implicated as regulators of RNA-containing condensates, controlling RNA content and enabling spatially focused control of RNA processing (52).

*T. brucei* progresses from the epimastigote in the tsetse fly salivary glands, to the metacyclic form preadapted for transmission, to the BSF. This involves initial expression of metacyclic *VSG* from MESs, after which monoallelic BSF *VSG* expression from a BES becomes established. It has been shown that premetacyclic cells express multiple *mVSG* transcripts, which is resolved to singular *mVSG* expression in mature metacyclic cells (53). Whether a similar “transcriptional race” among BESs occurs in BSF cells, or if the monoallelic expression has already been established for *mVSG* and a single BES activates by a switching mechanism remains to be determined. Notably, promoter competition is linked to chromosome X inactivation in females, a classical model of allelic exclusion (54). Transcriptomics of metacyclic and BSF stages (32, 35–37) indicates that ESB1 expression precedes ESBX. While this is RNA-level data without corresponding protein validation, the sequential expression pattern may reflect the temporal requirements for ESB assembly during metacyclic to BSF lifecycle transitions.

Our data support a model of distinct BES transcriptional states. First, the fully engaged state (FE state), characterized by processive transcription and high *VSG* to *ESAG* transcript ratios observed at the active BES. Second, a low-level trickle state (T state), characterized by promoter-proximal *ESAG* transcription (55), as observed at inactive BESs under normal conditions. Third, an enhanced T (ET) state observed when inactive BESs show increased promoter-proximal transcript beyond the typical T-state levels, as seen following VEX2 RNAi (25, 26), ESB1 overexpression, (28) and ESBX RNAi (Fig. 3).

The FE state experiences monoallelic restriction; the T/ET states, based on VEX2 RNAi (25, 26) and ESB1 overexpression (28) characterization, do not. This suggests multiple transcriptional states, although not necessarily physiological, can be achieved in BSFs. ET states are perhaps incapable of progressing to an FE state without the currently active BES leaving the FE state, as transcriptional machinery becomes limiting, consistent with recent observations of VEX2 knockdown using scRNA-Seq (26).

We propose a model where stochastic increase in transcription from a promoter in a T state leads to a cis-acting positive feedback through cotranscriptional recruitment of processing factors, potentially establishing an FE state at that BES. This may occur through sufficient nascent transcript recruitment of processing factors or through specialized RNAs encoded promoter-distally [like conserved *ESAG* pseudogenes or transcribed *VSG*-upstream repeats (4)]. Simultaneously, trans-acting effects may sequester transcription activating factors away from other BES promoters, establishing the monoallelic state. Testing this model will require determining the molecular mechanisms underlying factor recruitment and redistribution, including potential roles for protein complex assembly, ESBX co-operativity, or phase separation in ESB formation.

In conclusion, we show that Tb927.3.1660/ESBX is an ESB component required for integrating transcription and repression of antigen coding genes. It is required for transcription, but only of the active BES, and is required for maintaining the inactivity of inactive BESs. This is a link between BES transcription activation and inactive BES repression, allowing us to propose a model for establishment of monoallelic *VSG* expression.

## Methods

### *T. brucei* Growth and Genetic Manipulation.

*T. brucei* Lister 427 BSF cells were cultured in modified HMI-9 medium supplemented with 10% FCS at 37 °C with 5% CO_2_ and maintained below 2 × 10^6^ cells mL^−1^. For RNAi and overexpression experiments, 2T1 BSF cells were used (56). For CRISPR-Cas9 tagging experiments, we used the pSmOx cell line expressing spCas9 proteins (57, 58). Transfections were performed by electroporation with 10 μg of linearized plasmid DNA in Roditi Tb-BSF buffer (59). Clonal cell lines were generated by limiting dilution and drug selection.

### Proximity-Dependent Biotinylation.

To identify proteins proximal to ESB1, we used the S16 parental cell line (with puromycin resistance gene downstream of the *VSG221* BES1 promoter) (60) that also expressed Ty::Halo::RPA2 (Hygro) (44). ESB1 was fused to 6× HA mT and 3× Myc TurboID biotin ligases at the N- or C-terminus with epitope tags, generating four constructs which integrate at the endogenous ESB1 locus. Expression of the fusion proteins was confirmed by western blotting and immunofluorescence using anti-HA or anti-Myc antibodies, with RPA2 serving as an ESB reference marker.

### Biotinylation and Streptavidin Affinity Purification.

Experiments were carried out as previously described (29). Briefly, cells (5 × 10^8^) expressing the ESB1 fusion proteins were incubated with 50 μM biotin for 18 h. Cells were lysed in RIPA buffer, sonicated, and treated with micrococcal nuclease. Biotinylated proteins were affinity-purified using streptavidin beads under stringent conditions with appropriate parental and no-biotin controls. Eluted samples were verified by western blotting, and analyzed by mass spectrometry.

### Mass Spectrometry and Protein Identification.

Streptavidin beads with bound proteins were digested on bead, eluted, and analyzed by DIA-PASEF on a Bruker timsToF HT. The resulting LC–MS data were searched against the *T. brucei* proteome (Lister427- 2018, TriTrypDB version 68) database. Fold enrichment of each individual protein and statistical analysis were conducted using FragPipeAnalystR (61) without imputation. False discovery rate (FDR) was controlled at 1% using the Benjamini–Hochberg method.

### Plasmid Constructs: Endogenous Tagging, RNAi, and Exogenous Expression.

For C-terminal tagging of Tb927.3.1660 (ESBX) at its endogenous locus, a 464 bp CDS fragment lacking the stop codon and the first 473 bp of the 3’UTR were cloned into pEnT5^6×HA^ to add a C-terminal 6× HA tag. All constructs were linearized prior to transfection. Similar approaches were used to generate GFP-tagged VEX2, Halo-tagged RPA2, and ESB1 cell lines.

For ESBX RNAi, a 572 bp fragment of the Tb927.3.1660 ORF was selected using RNAit (62), cloned into the pRPaiSL vector (63), linearized with *Asc*I, and integrated into the 2T1 cell line landing pad. RNAi was induced with 1 μg mL^−1^ doxycycline. knockdown efficiency was monitored by immunofluorescence microscopy, western blotting, and RNA-seq was performed to determine transcript abundance.

For inducible exogenous expression of ESBX, the complete ORF with a C-terminal 6× HA tag was cloned into the pRPa tetracycline-inducible expression vector (63), the construct was integrated into the 2T1 landing pad (56), expression was induced with doxycycline and analyzed as by western blotting and immunofluorescence.

### Growth, Cell Cycle, and Western Blotting Analysis.

Cells were seeded at 1 × 10^5^ cells mL^−1^ and counted every 24 h using a hemocytometer. For RNAi and overexpression experiments, paired induced (1 μg mL^−1^ doxycycline) and uninduced cultures were maintained. Cell cycle analysis was performed by counting kinetoplasts and nuclei in DAPI-stained cells. At least 200 cells were analyzed per condition.

Whole cell lysates were separated by SDS-PAGE, transferred to PVDF membranes and probed with primary antibodies: anti-HA 3F10 (1:1,000, rat monoclonal, Roche) or anti-c-Myc (1:10,000, mouse monoclonal, clone 4a6, Merck Millipore). For detection of biotinylated proteins in proximity labeling experiments, membranes were incubated with streptavidin-HRP (1:10,000, Thermo Fisher) or HRP-conjugated secondary antibodies and chemiluminescent substrate.

### Immunofluorescence.

Cells were fixed with 4% methanol-free formaldehyde, permeabilized with 0.2% Triton-X100, and blocked in 2% BSA. Primary antibodies were rat anti-HA (3F10 Roche, 1:500) and rabbit anti-GFP (A11122 Invitrogen, 1:250). The secondary antibodies used were goat-anti-Rat IgG (H + L) conjugated to Alexa Fluor 488 or 594 (Invitrogen) and goat-anti-rabbit IgG (H + L) conjugated to Alexa Fluor 488. Halo::RPA2 or Halo::ESB1 tagged cells were labeled in vivo with 200 nM of JF571 HaloTag ligand (Janelia).

### Microscopy Quantitation.

ESBX foci signal intensity and full width half maximum (FWHM) signal were measured by fitting a Gaussian $y=a+\left(b-a\right)e^{-{\left(x-c\right)}^{2}/2d^{2}}$ to mean signal intensity in the horizontal direction across a 20 × 80 pixel selection centered on the focus, using the ImageJ get profile and curve fitting tools. *b* was taken as the signal intensity, FWHM $2\sqrt{2\ln2d^{2}}$ was calculated from the SDd. Data points with poor fit ($R^{2}<0.9$), c out of the analyzed area or with an outlier d>4 pixels were excluded. FWHM was compared to that from TetraSpeck 0.1 μm multicolor fluorescent beads (ThermoFisher) and the theoretical FWHM of the Airy disk of 0.8038×1.22λ/NA which, for λ = 520 and NA = 1.4, is 183.1 nm.

Distance between ESB1, ESB RPA2, and ESBX signal foci were measured using the same method we previously used to analyze ESB1 (28). Using ImageJ, we used a 5 pixel rolling ball filter to subtract background signal then fitted a Gaussian to the mean signal intensity in the horizontal and vertical direction across a 10 × 10 pixel selection centered on the green channel focus. Foci with a poor fit in either the red or green channel, $R^{2}<0.95$ or c out of the analyzed area, were excluded. To correct for chromatic aberration, correlation of distance between red and green foci and position in the image in the horizontal and vertical directions was measured using TetraSpeck 0.1 μm multicolor fluorescent beads (ThermoFisher). A linear fit of distance and position was used to generate a linear correction in the horizontal and vertical direction, which was applied to all measurements.

### RNA Sequencing Analysis.

Three independent clones of ESBX::6× HA/ESBX RNAi cell lines were analyzed alongside parental controls. Total RNA was purified using the Qiagen RNeasy Plus Mini Kit, cDNA was generated using reverse transcription and a poly-dT primer for poly-A selection, then sequenced using 100 bp read length 200 bp insert size paired end sequencing (BGISEQ-500) and >50 million reads per sample.

To quantify transcript abundance, sequencing reads were aligned to the predicted transcriptome of the *T. brucei* Lister 427 2018 genome (7) [from TriTrypDB version 68 (31)] augmented with the 5′ and 3′ UTRs we previously mapped (28) (https://zenodo.org/records/17872563), enabling quantitation from the whole transcript rather than just the predicted CDSs for most genes. Reads were aligned to the predicted transcriptome using BWA-MEM with default settings, then filtered to only uniquely mapped reads using samtools view with the command line flags -q 10, -F 0x504 and -f 0x02. This is exactly as we previously described for analysis of ESB1, where we showed that this filtering gives >99.75% accurate mapping of reads to ESAGs in specific BESs (28). Reads per kilobase per million reads (RPKM) per transcript was calculated from the output from samtools idxstats. Two methods were used to calculate statistical significance: using the two-tailed *t* test of log-transformed RPKM of the three induced to three uninduced samples, and using EdgeR v4 with FDR-correction (64).

Unprocessed (immature or nascent) transcripts were quantified by filtering for only sequencing reads spanning a polyadenylation or spliced leader acceptor site (PAS or SLAS), weighted by the frequency of use of that site, using the PASs and SLASs sites and frequency we previously determined for analysis of ESB1 (28). PAS and SLAS usages are reported as site usage-weighted mapped reads spanning a site, normalized per million mapped reads (RPM) (28).

Inactive and active BES *ESAGs* and *VSGs* and MESs were identified from *T. brucei* Lister 427 2018 genome annotation (7) having confirmed BES1 is active. For plotting, all *VSG* transcript sets were defined by BLASTn search using each VSGnome (6) as query sequences, accepting hits at least 500 nucleotides long, at least 50% of the query sequence, and at least 50% identity with the query. Distance to a BES promoter was taken as the distance to the closest upstream promoter.

### Key Resources.

All plasmids, cell lines, antibodies, primers, and key reagents used in this study are listed in Dataset S4. Detailed experimental protocols are provided in *SI Appendix*.

## Supplementary Material

Appendix 01 (PDF)

Dataset S01 (XLSX)

Dataset S02 (XLSX)

Dataset S03 (XLSX)

Dataset S04 (XLSX)

## Data Availability

Proteomics data have been deposited in MassIVE (65) and ProteomeXchange (66). RNA-seq data have been deposited in European Nucleotide Archive (67). Other data are included in the manuscript and/or supporting information.
