# Supplementary material for: A factor integrating transcription and repression of surface antigen genes in African trypanosomes
Source: Proc Natl Acad Sci U S A. 2026 Feb 3;123(6):e2531377123. doi: 10.1073/pnas.2531377123 (PMC12890818; doi:10.1073/pnas.2531377123)
Supplement: Supplementary file 1 — Appendix 01 (PDF) [file pnas.2531377123.sapp.pdf]

Supporting information for

A novel factor integrating transcription and repression of surface antigen genes in African trypanosomes

María Agustina Berazategui[1], Ione Goodwin[1], Lianne I M Lansink[2], Keith Gull[1,3], Gloria Rudenko[1†], Jack D Sunter[4*], Joana R C Faria[2*], Richard J Wheeler[5*], Calvin Tiengwe[1*]

1. Department of Life Sciences, Imperial College London SW7 2AZ, London, UK
2. Department of Biology, University of York, York YO10 5DD, UK
3. Sir William Dunn School of Pathology, University of Oxford OX1 3RE, Oxford, UK
4. Department of Biological and Medical Sciences, Oxford Brookes University, Oxford OX3 0BP, UK
5. School of Biological Sciences, Institute for Immunology and Infection Research, University of Edinburgh, Edinburgh EH9 3FL, UK

† Deceased 24.11.2022.

*To whom correspondence should be addressed

Corresponding authors: jsunter@brookes.ac.uk, joana.correiafaria@york.ac.uk, richard.wheeler@ed.ac.uk, c.tiengwe@imperial.ac.uk

This PDF file includes:

Datasets S1 to S4
Figures S1 to S5
Detailed materials and methods
SI References

Dataset S1: Summary of hits from proximity-dependent labelling
List of 26 candidate proteins identified by BioID using ESB1 as bait, following bioinformatic filtering of the complete mass spectrometry dataset. Cells expressing ESB1 chimeric proteins were incubated with biotin, lysed and whole cell extracts were precipitated using streptavidin beads, and analyzed by LC-MS/MS (n = 2 technical replicates per cell line). Mean Log2 fold change (Log_2FC) values from duplicate experiments compare N-terminal (6×HA::mT::N×2, TID::N×1) and C-terminal (mT::HA C×2, TID::3×Myc C×1) fusions to parental controls. Green shading indicates significant enrichment thresholds (dark green Log_2FC >3; light green Log_2FC <3). Gene annotations and domain predictions are derived from TriTrypDB release 68. Complete proteomics dataset with all detected proteins and statistical values is provided in **Dataset S3**.

Dataset S2: Bioinformatics screen
In silico screen produced 175 candidate genes, winnowed by biochemical plausibility to 79 genes, refined by subcellular localisation, if known, to 48 genes.

Dataset S3: Complete proteomics and RNA sequencing datasets
Sheet 1: Full proteomics data from proximity-dependent biotinylation experiments showing log2 fold changes and *p*-values for all detected proteins across four ESB1 fusion constructs (2×mT.ESB1, 2×ESB1.mT, 1×TurboID.ESB1, 1×ESB1.TurboID).

Sheet 2: Complete RNA-Seq data from ESBX RNAi experiments (12h and 24h post-induction) and ESBX overexpression experiments (24h post-induction) showing mean RPKM values, log2 fold changes, and *p*-values using two-tailed t tests on log-transformed RPKM for all transcripts.

Sheet 3: EdgeR differential expression of RNA-seq data from ESBX RNAi experiments (12h and 24h post-induction) and ESBX overexpression experiments (24h post-induction) showing log2 fold change (logFC), log2 counts per million (logCPM), and FDR-adjusted *p*-values for all transcripts.

Sheet 4: Read counts spanning spliced leader acceptor and polyadenylation sites from ESBX RNAi experiments (12h and 24h post-induction) and ESBX overexpression experiments (24h post-induction).

Dataset S4: Antibodies, plasmids, cell lines and primers

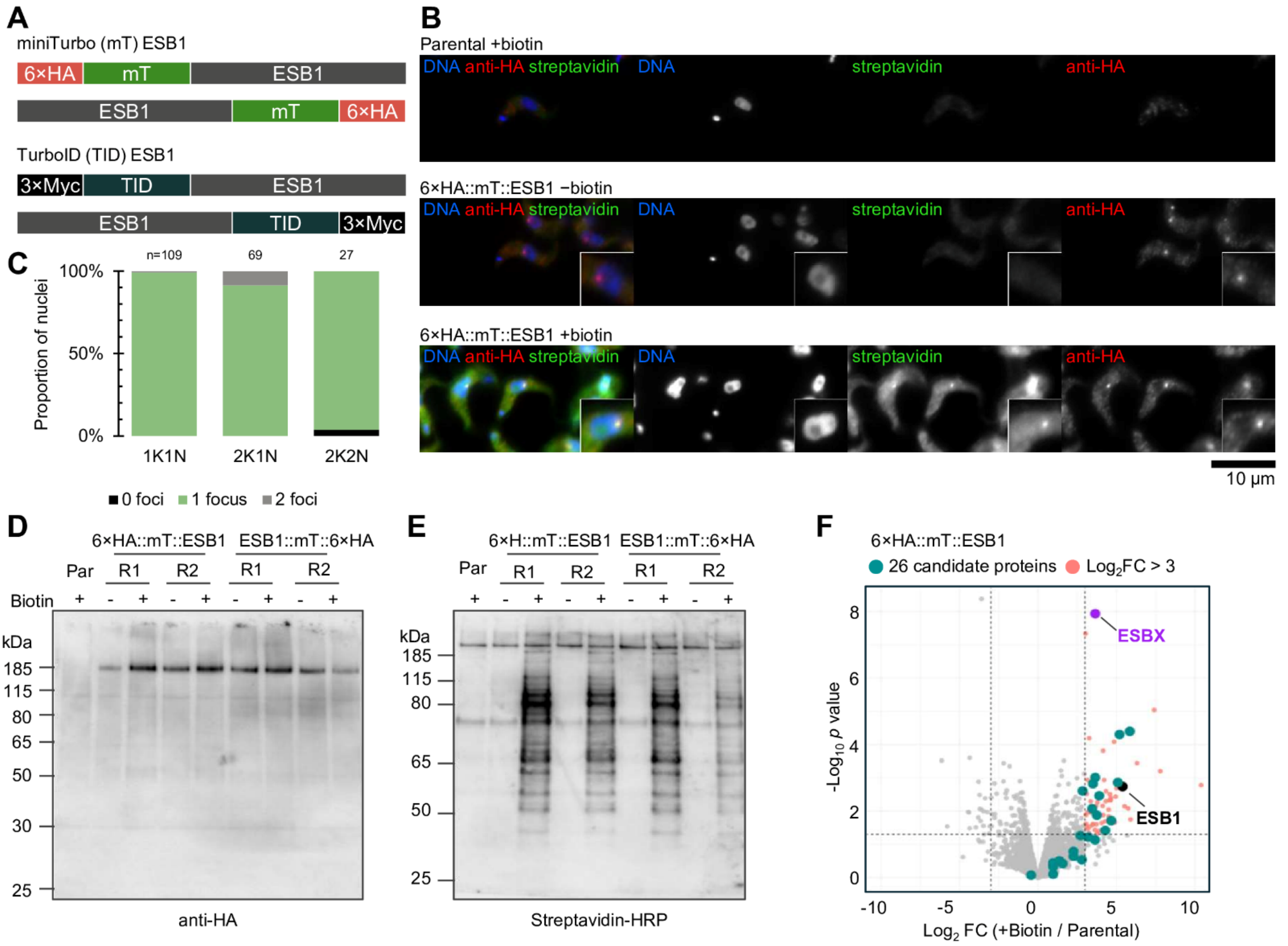

Figure S1. Proximity-dependent biotinylation screen to identify proteins near ESB1

A. Schematic of ESB1 fusion constructs with two different biotin ligases. The constructs show ESB1 fused at the 5′ or 3′-end to either miniTurbo (mT) or TurboID (TID), with 6×HA or 3×Myc epitope tags respectively.

B. Subcellular localization of 6×HA::mT::ESB1. Parental cells expressing Halo::RPA2 and cells expressing both Halo::RPA2 and 6×HA::mT::ESB1 were incubated without or with biotin (50 μM, 18 h), fixed, permeabilised, and co-stained. Biotinylated proteins were detected with streptavidin-Alexa Fluor 488; ESB1 chimeric protein with anti-HA primary antibody/Alexa Fluor 594. Representative images show merged and individual channels: DNA (Hoechst, blue), biotinylated proteins (streptavidin-Alexa Fluor 488, green), and 6×HA::mT::ESB1 (anti-HA, red). Scale bars = 10 µm. Insets show magnified views of a single nucleus. Similar localization patterns were observed for ESB1-TID and C-terminal ESB1-mT expressing cell lines.

C. Quantitative analysis of 6×HA::mT::ESB1 nuclear foci distribution across cell cycle stages showing the proportion of foci/nuclei containing 0 (black), 1 (green), 2 (grey) foci. Numbers above bars indicate total nuclei counted (n) for each cell cycle stage.

D, E. Western blot analysis of 6×HA::mT::ESB1 expression and biotinylation efficiency. Cell lysates from parental (Par) and 6×HA::mT::ESB1-expressing cell lines (R1, R2 = replicates 1 and 2) were treated with (+) or without (-) biotin (50 µM, 18 h). 6×HA::mT::ESB1 protein was detected using anti-HA antibody **(D),** and biotinylated proteins were detected using streptavidin-HRP (**E**), Molecular weight markers (kDa) are shown.

F. Volcano plot showing protein enrichment in biotinylated samples relative to parental controls for 6×HA::mT::ESB1 fusion construct. Each dot represents a protein, and green dots show the 26 candidate proteins in Dataset S1 with $Log_2FC > 3$ in at least one experiment. Red dots indicate all proteins with $Log_2FC > 3$. ESBX and ESB1 are highlighted separately.

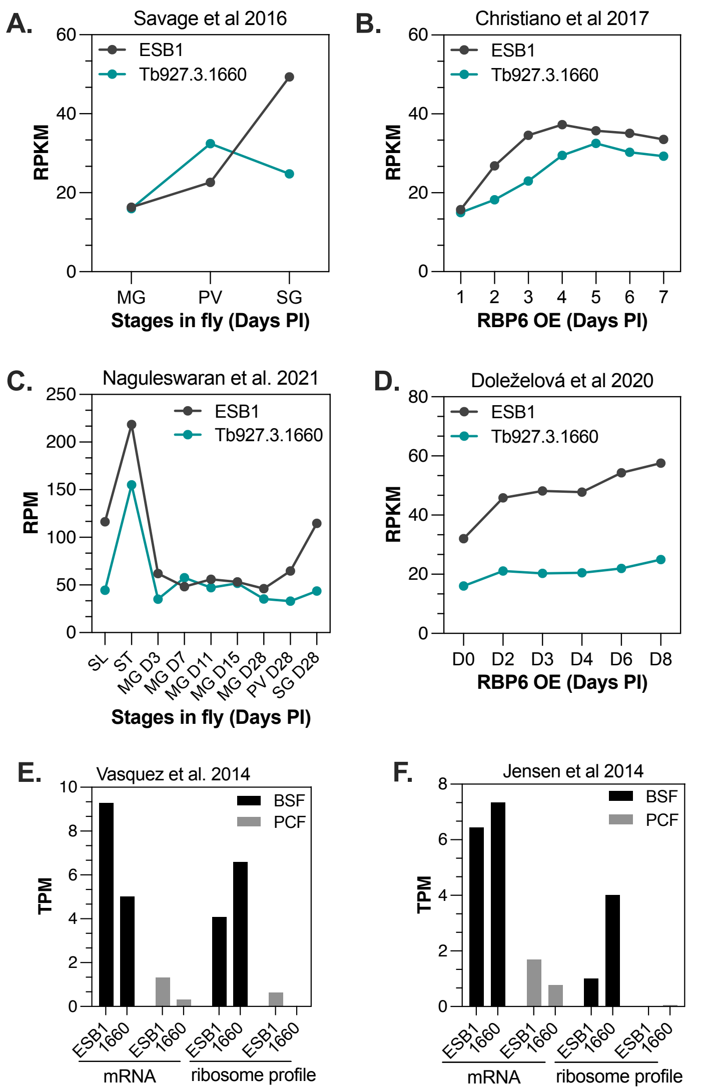

Figure S2. Comparative analysis of transcript expression profiles ESB1 and Tb927.3.1660 across different developmental stages of *T. brucei*.

Each panel shows transcript expression levels (RPKM/RPM/TPM) of ESB1 (black lines) and Tb927.3.1660 (green lines) genes across different developmental stages or experimental conditions.

A. Expression patterns during fly developmental stages (MG, midgut; PV, proventriculus; SG, salivary gland) from Savage et al. 2016[1] show increased ESB1 but not Tb927.3.1660 expression in SG.

B. Expression profiles following RBP6 overexpression (OE) over 7 days post-induction (Days PI) from Christiano et al. 2017[2], showing ESB1 expression increases before Tb927.3.1660.

C. Expression dynamics throughout developmental stages (SL, slender bloodstream; ST, stumpy bloodstream) from Naguleswaran et al. 2021[3], showing regulation across multiple time points and tissues. Much lower ESB1 and Tb927.3.1660 expression in MG forms, and increased ESB1 but not Tb927.3.1660 expression in SG.

D. Expression patterns during RBP6 overexpression from Doleželová et al. 2020[4], showing increase in ESB1 prior to Tb927.3.1660 .

E,F. Transcript level comparisons between bloodstream forms (BSF) and procyclic forms (PCF) from Vasquez et al. 2014[5] (**E**) and Jensen et al. 2014[6] (**F**), showing both mRNA abundance and ribosome occupancy data. Both datasets show higher ESB1 and Tb927.3.1660 (1660) mRNA expression in BSF compared to PCF. The low mRNA levels and absence of ribosome-associated transcripts of Tb927.3.1660 in PCF indicate that the protein is likely not translated in this lifecycle stage.

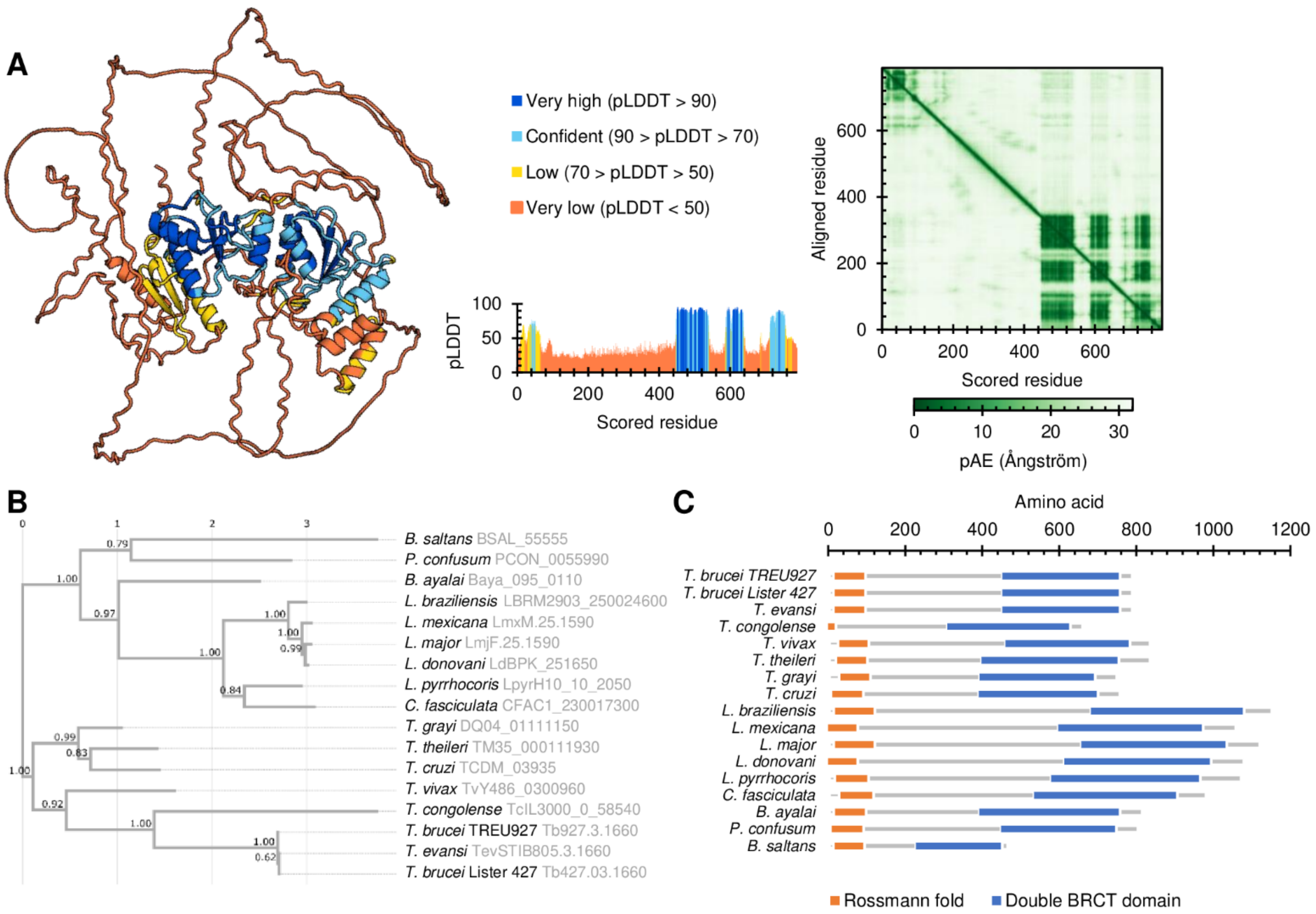

Figure S3. ESBX encodes a trypanosomatid-specific BRCT domain-containing protein.

A. AlphaFold2-predited structure of ESBX. Left, the structure, coloured by predicted local distance difference test (pLDDT). Centre, a plot of pLDDT by residue. Right, a plot of pairwise predicted average error (PAE) in residue position. There is a predicted N-terminal Rossman fold structured domain and large C terminal double BRCT-like structured domain.

B. Phylogenetic tree of the protein sequence of ESBX and its orthologs among kinetoplastids.

C. Representation of the linear protein sequence of ESBX and its orthologs among kinetoplastids, indicating the position of the structured domains. The *T. congolense* ortholog gene model likely has an incorrectly called start codon.

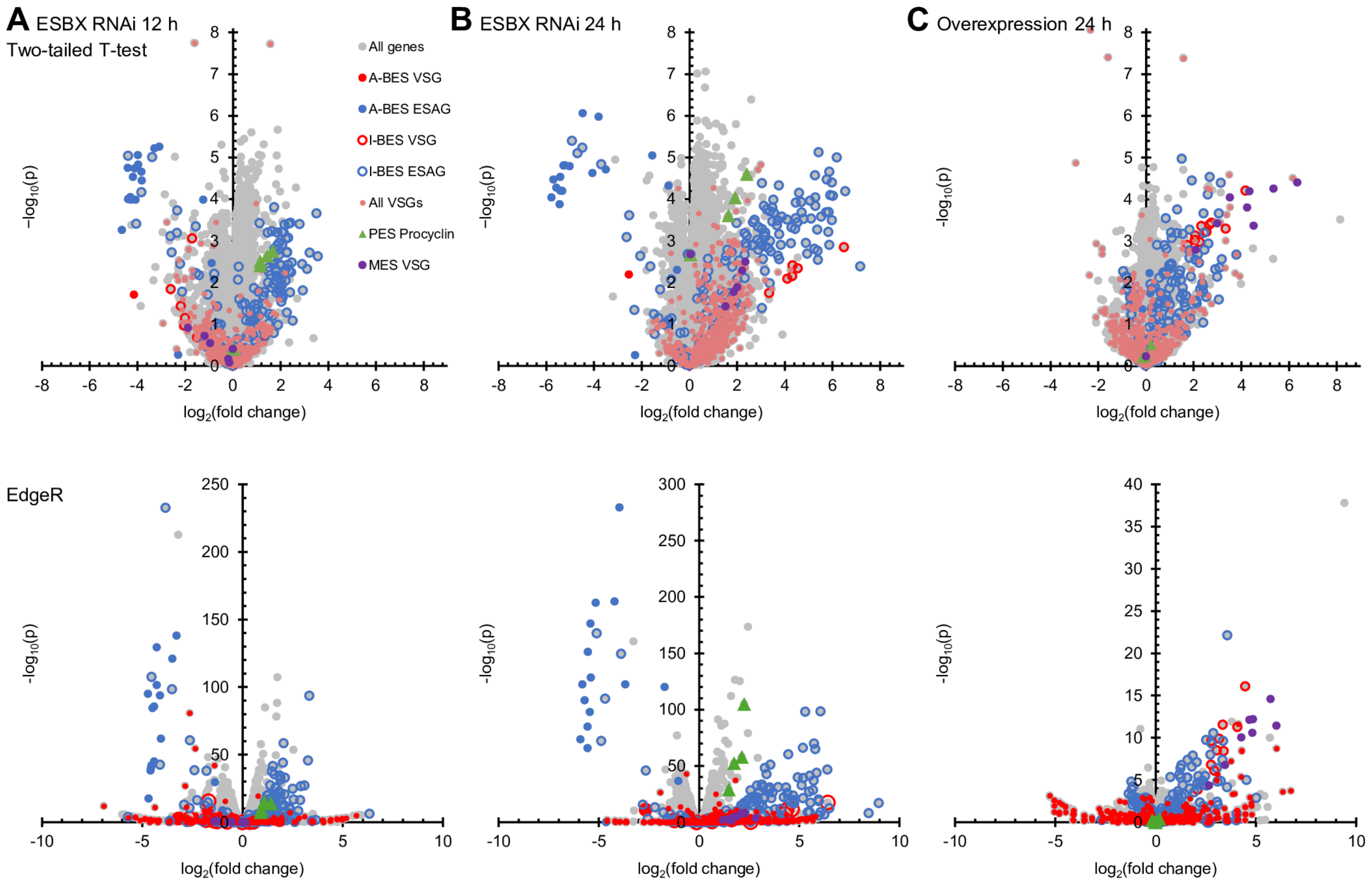

Figure S4. Volcano plot representation of RNAseq data

Plot of fold-change plotted against *p* value for (top) two tailed t-test of log RPKM without multiple comparison correction and (bottom) using EdgeR with FDR correction of induced relative to uninduced sample for:

A. ESX RNAi, 12 h after induction, three independent clones. Alternative plot of data shown in **Fig 3A**.

B. ESBX RNAi, 24 h after induction, three independent clones. Alternative plot of data shown in **Fig 3E**.

C. ESBX overexpression, 24 h after induction of exogenous expression, three replicates of one representative clone. Alternative plot of data shown in **Fig 4F**.

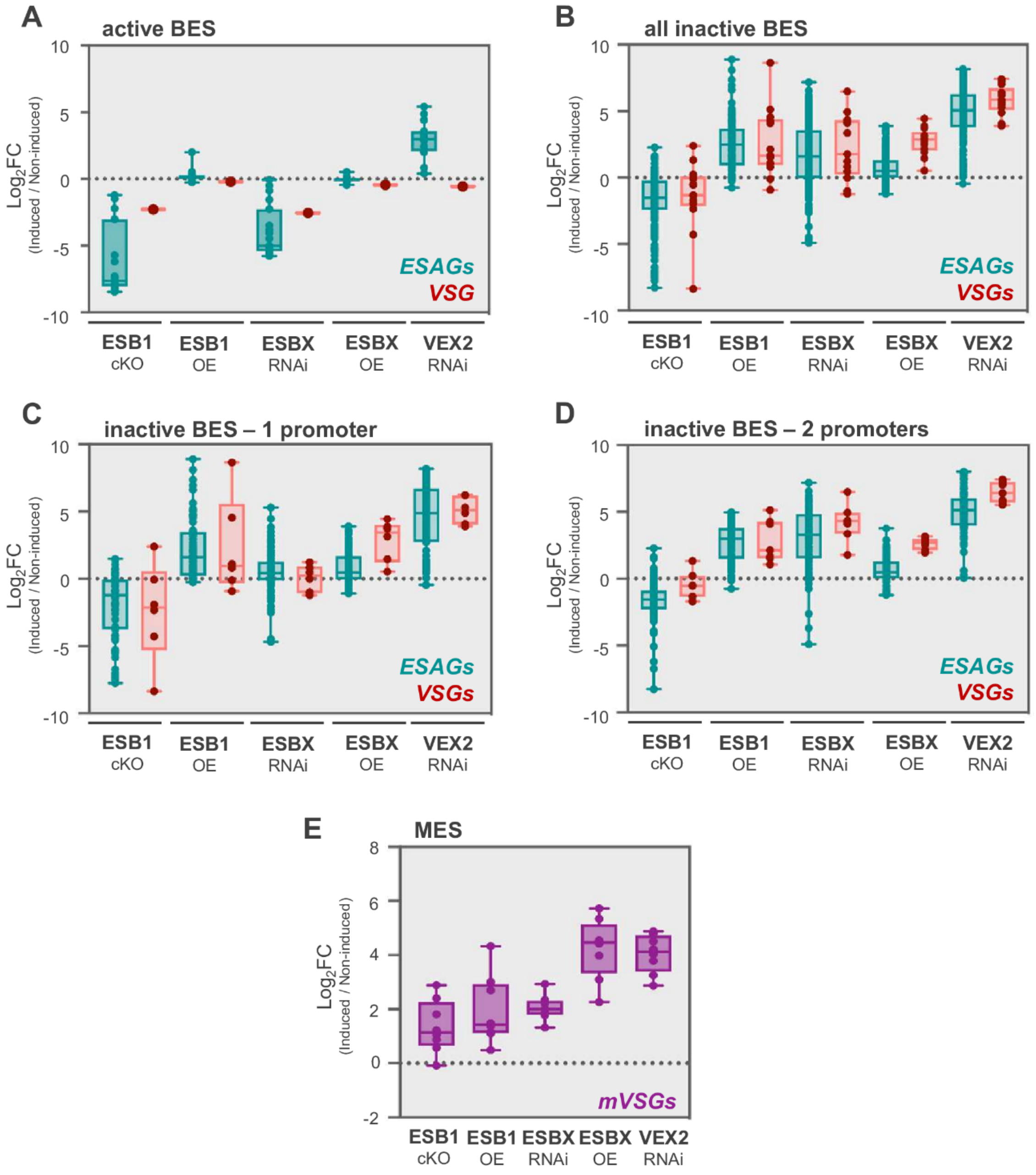

Figure S5. Comparative RNA-Seq analysis between ESB1, ESBX and VEX2 depletion and overexpression datasets with a focus on BES and MES transcripts.

Boxplots depict fold changes in transcript abundance, expressed as Log2FC, between induced and non-induced samples (24 h post induction; except ESB1 cKO – 48 h) for transcripts originated from the following Pol-I transcription units:

A. active BES

B. all inactive BES

C. inactive BES containing 1 Pol-I promoter

D. inactive BES containing 2 Pol-I promoters

E. MES.

The boxes extend from the 25th to the 75th percentile; the line represents the median value; whiskers span between minimum and maximum values. All datapoints are displayed and represent individual transcripts; values are averages of three independent biological replicates. cKO (conditional knock-out); OE (overexpression). Previously published RNA-Seq datasets for VEX2 RNAi[7] (PRJEB21615) and ESB1 cKO and OE[8] (PRJNA784098) were reanalyzed using an identical pipeline to the ESBX analysis described here.

Detailed materials and methods

T. brucei growth and genetic manipulation

T. brucei Lister 427 bloodstream form (BSF) cells were cultured in modified HMI-9 medium (Thermo; 07490915 N) supplemented with 10% FCS (Gibco; 10270106) and 0.2 mM β-mercaptoethanol (Sigma; 63689) at 37°C with 5% CO_2, maintained at densities below 2 × 10^6 cells mL^{-1} by regular subculture. The active bloodstream expression site (BES) was BES1 containing *VSG221* (*VSG 427-2*). For RNAi and overexpression experiments, 2T1 BSF cells were used[9], which contain a tetracycline-inducible system integrated at a single genomic locus for robust inducible expression. For CRISPR-Cas9 tagging experiments, we used the pSmOx cell line expressing T7 RNA polymerase, Tet repressor and spCas9 proteins[10,11].

Transfections were performed by electroporation using the Amaxa Nucleofector IIb (Lonza, program X-001) with 10 µg of linearised plasmid DNA in Roditi Tb-BSF buffer[12] with 3 × 10^7 cells. After electroporation, cells were transferred to 10 ml of pre-warmed medium for 6 h before adding selection drugs. Drug concentrations for selection were: 0.2 µg mL^{-1} puromycin dihydrochloride, 5.0 µg mL^{-1} blasticidin S hydrochloride, 2.0 µg mL^{-1} G-418 disulfate, 5 µg mL^{-1} hygromycin B, and 2.5 µg mL^{-1} phleomycin. Clonal cell lines were generated by limiting dilution, with three independent clones typically selected for experimental analysis.

Proximity-dependent biotinylation

To identify proteins proximal to ESB1, we used the parental cell line (S16 clone containing a puromycin resistance gene downstream of BES1 promoter)[13] that also expressed Ty::Halo::RPA2 (Hygro)[14] as our background strain. Expression constructs were designed to express ESB1 fused at either the N- or C-terminus with biotin ligases miniTurbo (mT) and TurboID, along with epitope tags. Four constructs were generated: (1) 6×HA::mT::ESB1 (N-terminal miniTurbo, both alleles targeted), (2) TID::3×Myc::ESB1 (N-terminal TurboID, single allele targeted), (3) ESB1::mT::6×HA (C-terminal miniTurbo, both alleles targeted), and (4) ESB1::TID::3×Myc (C-terminal TurboID, single allele targeted).

For each construct, the appropriate targeting sequences were amplified from *T. brucei* 427 genomic DNA using Q5 DNA polymerase (NEB). For N-terminal tagging, the first 414 bp of the coding sequence (after the ATG) and the first 307 bp of the 5'UTR were used for targeting. For C-terminal tagging, the last 414 bp of the CDS (excluding the stop codon) and the first 307 bp of the 3'UTR were used. For the TurboID constructs, ESB1 targeting sequences were cloned into pNAT N/C – TurboID::3myc plasmids[15], where the TurboID sequence was codon optimised for expression in *T. brucei*. All constructs included a glycine-serine spacer of at least 24 nucleotides between the epitope tag and ESB1. All constructs were integrated at the endogenous ESB1 locus, and expression of the fusion proteins was confirmed by Western blotting. Subcellular localization was verified by immunofluorescence using anti-HA or anti-Myc antibodies, with RPA2 as a reference marker for the ESB.

Biotinylation and streptavidin affinity purification

Experiments were carried out as we previously described[15]. Briefly, BSF cells (5 × 10^8) expressing the ESB1 fusion proteins were seeded at 1 × 10^5 cells mL^{-1} in T175 flasks (two flasks per replicate) and incubated with 50 µM biotin for 18 h. Untagged parental Halo::RPA2-expressing cells treated with biotin served as controls, and tagged cell lines without biotin (with equivalent DMSO) were included as additional controls. Cells were harvested by centrifugation at 1,000 x g for 10 min at room temperature, washed twice in pre-warmed PBS/5% glucose and once in PBS. Cell pellets were transferred to low protein binding tubes before the final wash.

Cell pellets were lysed in 1 ml ice-cold RIPA buffer (50 mM Tris-HCl pH 7.4, 150 mM NaCl, 1% NP-40, 0.5% sodium deoxycholate, 0.1% SDS) supplemented with protease inhibitors (100 µl PMSF 100 mM, 77 µl Pepstatin 2 mM, 100 µl TLCK 0.1 M, 1 ml Proteoloc, and 5 tablets Roche Protease Inhibitor Cocktail plus 5 tablets Phosphostop per 100 ml buffer). Lysates were incubated on ice for 30 min, then transferred to pre-chilled Bioruptor tubes and sonicated using a Pico Bioruptor (3 cycles, 30 sec on/30 sec off, high settings). Micrococcal nuclease (1 µl, NEB) was added to each lysate for 10 min at room temperature followed by 50 min on ice. Lysates were clarified by centrifugation at 10,000 g for 10 min at 4°C.

For affinity purification, 100 µl of magnetic streptavidin bead suspension (Resyn Bioscience, 1 mg beads per sample) was transferred to 2 ml low protein binding tubes and washed twice with RIPA buffer using a magnetic rack. Clarified lysates (approximately 1 ml) were added to the beads and incubated at 4°C overnight with end-over-end rotation.

After overnight incubation, beads were washed with 1 ml of each buffer for 5 min with gentle mixing by inversion at the 2.5-minute mark: RIPA buffer (6 washes), 4 M urea in 50 mM ammonium bicarbonate pH 8.0 (2 washes), 6 M urea in 50 mM ammonium bicarbonate pH 8.0 (2 washes), and 1 M KCl in 50 mM ammonium bicarbonate pH 8.0 (2 washes). All washes were performed at 4°C using ice-cold buffers. Beads were finally resuspended in 500 µl of 50 mM ammonium bicarbonate pH 8.0.

For Western blot analysis, 40 µl of beads from each sample were separated using a magnetic rack, resuspended in 25 µl of Western blot sample buffer (71 µl Roche protease inhibitor cocktail, 12.5 µl β-mercaptoethanol, 416.5 µl LDS 4× buffer), heated at 70°C for 10 min, and the supernatant collected after magnetic separation. The remaining beads were stored at-80°C for mass spectrometry analysis.

Mass spectrometry and protein identification

Streptavidin beads with bound proteins were submitted to the proteomics facility (University of York) where samples were digested on bead and 65% of each sample was loaded onto an EvoTip Pure for introduction onto a 15 cm endurance column. Elution was carried out using a 30 SPD pre-set gradient on an EvoSep One UPLC system. DIA-PASEF data were acquired using a Bruker timsToF HT with a 1.1 s cycle time, between 400-1200 m/z and using 25 m/z DIA windows.

Resulting LC-MS data in Bruker .d format were searched in DIA-NN against the *T. brucei* proteome (Lister427-2018, TriTrypDB version 68) database. An in-silico predicted spectral library was created with the DIA-NN software, which was iterated against the DIA data generated for these samples. The search was run at 1% FDR. The DIA-NN .tsv output was compiled, filtered (two peptide minimum) and pivoted to a protein group-centric output using custom KNIME pipelines.

Fold enrichment of each individual protein in +biotin samples *versus* the parental cell line control as well as statistical analysis were conducted using FragPipeAnalystR[16] without imputation. False Discovery Rate (FDR) correction was performed using the Benjamini-Hochberg method.

Plasmid constructs: endogenous tagging

For C-terminal tagging of Tb927.3.1660 (ESBX) at its endogenous locus, the last 464 bp of the CDS lacking the stop codon and the first 473 bp of the 3'UTR were PCR-amplified and cloned into pEnT5$^{6\times HA}$ to add a C-terminal 6×HA tag. Constructs were linearised with an appropriate restriction enzyme (between the cloned fragments) prior to transfection. Similar approaches were used to generate GFP-tagged VEX2, Halo-tagged RPA2 and ESB1 cell lines.

Plasmid constructs: RNAi

For inducible RNAi knockdown of ESBX, a 572 bp fragment of the Tb927.3.1660 ORF was selected using RNAit[62] and BLAST analysis to minimise potential off-target effects. This fragment was amplified by PCR using Q5 DNA polymerase (NEB) and cloned into pRPaiSL, a doxycycline-inducible RNAi vector[17]. The pRPaiSL construct contains two copies of the target fragment in reverse complement orientation separated by a 486 bp stuffer sequence, creating a stem-loop structure when transcribed. A rDNA promoter under the control of doxycycline drive transcription of this stem-loop. The construct was linearized with *Asc*I before transfection, targeting integration into the landing pad integrated at a rDNA spacer of the 2T1 cell line. RNAi was induced with 1 µg mL^{-1} doxycycline. To confirm effective knockdown, cell lines were generated containing both the RNAi construct and an endogenously tagged version of ESBX. Knockdown efficiency was monitored by immunofluorescence microscopy (confirming expected increase in 2K2N cells 12 h post-induction), Western blotting, and RNAseq were performed to determine transcript abundance.

Plasmid constructs: exogenous expression

For inducible exogenous expression of ESBX, the complete ORF with a C-terminal 6×HA tag was cloned into the pRPa tetracycline-inducible expression vector[17], the construct was excised with *Asc*I before transfection and integrates into the landing pad tagged ribosomal DNA locus of 2T1 cells[9]; expression was induced with 1 µg mL^{-1} doxycycline.

Growth and cell cycle analysis

Cells were seeded at 1×10^5 cells mL^{-1} and counted every 24 h using a haemocytometer. Cultures were diluted to 1×10^5 cells mL^{-1} during each count to maintain continuous growth. For RNAi and overexpression experiments, paired induced (1 µg mL^{-1} doxycycline) and uninduced cultures were maintained. Cell cycle analysis was performed by counting kinetoplasts and nuclei in DAPI-stained cells. At least 200 cells were analyzed per condition.

Western blotting

Whole cell lysates were prepared from 1×10^7 cells by resuspending cell pellets in SDS-PAGE sample buffer (3× sample buffer with 50 mM DTT final concentration) and immediately boiling for 10 minutes at 70°C. Samples were vortexed and centrifuged briefly before loading. Proteins were separated on 10% Tris-Glycine at 100 V for 1.5 hours until the loading dye reached the bottom of the gel.

Proteins were transferred to PVDF membranes using a Trans-Blot Turbo Transfer System (Bio-Rad) at 2.5 A, 25 V for 10 minutes. Membranes were blocked with 5% milk in PBS-Tween (PBS with 0.1% Tween-20) for 1 hour at room temperature, then incubated with primary antibodies diluted in 2% milk in PBS-Tween for 1 hour: anti-HA 3F10 (1:1000, rat monoclonal, Roche) or anti-c-Myc (1:10, 000, mouse monoclonal, clone 4a6, Merck Millipore). After three 5-minute washes with PBS-Tween, membranes were incubated with HRP-conjugated secondary antibodies (goat-anti-mouse HRP, 1:10,000 in 5% milk) for 1 hour at room temperature.

Following three 5-minute washes with PBS-Tween, signals were detected using SuperSignal West Pico Chemiluminescent Substrate (Thermo Scientific) and imaged using a ChemiDoc transilluminator (Bio-Rad). For loading controls, stain-free imaging of SDS-PAGE gels was performed using a ChemiDoc transilluminator.

For detection of biotinylated proteins in proximity labelling experiments, membranes were incubated with streptavidin-HRP (1:10,000, Thermo Fisher) for 1 hour after blocking with 5% BSA, followed by washing and detection as described above.

Immunofluorescence

Cells were fixed in media using a final concentration of 4% methanol-free formaldehyde for 10 min, washed twice in 1× PBS and settled on slides for 1h. Cells were permeabilised with 0.2% Triton-X100 for 10 min, washed three times with 1× PBS and blocked in 2% BSA. Subsequently, cells were incubated with following primary antibodies: rat anti-HA (3F10 Roche, 1:500) and rabbit anti-GFP (A11122 Invitrogen, 1:250). The secondary antibodies used were goat-anti Rat IgG (H+L) conjugated to Alexa Fluor 488 or 594 (Invitrogen) and goat-anti rabbit IgG (H+L) conjugated to Alexa Fluor 488.

Cells with endogenously tagged Halo::RPA2 or Halo::ESB1 were labelled *in vivo* by adding a final concentration of 200 nM of JF571 HaloTag ligand (Janelia) to the media. Cells were incubated for 30 min at 37°C and fixed as described above.

Microscopy quantitation

ESBX foci signal intensity and full width half maximum (FWHM) signal were measured by fitting a Gaussian $y = a + (b - a)e^{-(x-c)^2/2d^2}$ to mean signal intensity in the horizontal direction across a 20 × 80 pixel selection centred on the focus, using the ImageJ get profile and curve fitting tools. b was taken as the signal intensity, FWHM $2\sqrt{2 \ln 2\, d^2}$ was calculated from the standard deviation d. Data points with poor fit ($R^2 < 0.9$), c out of the analyzed area or with an outlier $d > 4$ pixels were excluded. FWHM was compared to that from TetraSpeck 0.1 µm multi-colour fluorescent beads (ThermoFisher) and the theoretical FWHM of the Airy disk of $0.8038 \times 1.22\lambda/NA$ which, for λ = 520 and NA = 1.4, is 183.1 nm.

Distance between ESB1, ESB RPA2 and ESBX signal foci were measured using the same method we previously used to analyse ESB1[28]. Using ImageJ, we used a 5 pixel rolling ball filter to subtract background signal then fitted a Gaussian to the mean signal intensity in the horizontal and vertical direction across a 10 × 10 pixel selection centred on the green channel focus. Foci with a poor fit in either the red or green channel, $R^2 < 0.95$ or c out of the analyzed area, were excluded. To correct for chromatic aberration, correlation of distance between red and green foci and position in the image in the horizontal and vertical directions was measured using TetraSpeck 0.1 µm multi-colour fluorescent beads (ThermoFisher). A linear fit of distance and position was used to generate a linear correction in the horizontal and vertical direction, which was applied to all measurements.

RNA sequencing analysis

For RNAi experiments, three independent clones of ESBX::6×HA/ESBX RNAi cell lines were analyzed alongside parental controls. Cells were seeded at 1.5×10^5 cells mL^{-1} and samples were collected at 12h and 24h post-induction with 1 µg mL^{-1} doxycycline. For each timepoint, 1×10^8 cells were harvested by centrifugation at 3200g for 90s, washed once in serum-free HMI-9, and pelleted at 10,000g for 30 seconds. Cell pellets were immediately resuspended in 600 µl lysis buffer and total RNA was purified using the Qiagen RNeasy Plus Mini Kit according to the manufacturer's instructions. RNA was eluted in 30 µl nuclease-free water. RNA quality was assessed using a Bioanalyzer (Agilent) and samples with RIN > 8 were selected for library preparation.

Additional samples from each culture were collected for Western blot and immunofluorescence microscopy analyses to confirm efficient protein depletion. Kinetoplasts (K) and nuclei (N) were counted from DAPI-stained cells as a measure of cell cycle stage. Cells were classified as 1K1N (G1), 2K1N (G2), or 2K2N (post-mitotic) based on K and N counts.

For transcriptome sequencing, cDNA was generated using reverse transcription and a poly-dT primer for poly-A selection, then sequenced using 100 bp read length 200 bp insert size paired end sequencing (BGISEQ-500) and >50 million reads per sample.

To quantify transcript abundance, sequencing reads were aligned to the predicted transcriptome of the *T. brucei* Lister 427 2018 genome[7] (from TriTrypDB version 68[18]) augmented with the 5′ and 3′ UTRs we previously mapped[8] (https://zenodo.org/records/17872563), enabling quantitation from the whole transcript rather than just the predicted CDSs for most genes. Reads were aligned to the predicted transcriptome using BWA-MEM with default settings, then filtered to only uniquely mapped reads using samtools view with the command line flags-q 10,-F 0x504 and-f 0x02. This is exactly as we previously described for analysis of ESB1, where we showed that this filtering gives >99.75% accurate mapping of reads to *ESAGs* in specific BESs[8]. Reads per kilobase per million reads (RPKM) per transcript was calculated from the output from samtools idxstats. Two methods were used to calculate statistical significance: using two-tailed t-test of log-transformed RPKM of the three induced to three uninduced samples, and using the standard EdgeR v4 FDR-corrected significance calculation[64].

Unprocessed (immature or nascent) transcripts were quantified by filtering for only sequencing reads spanning a polyadenylation or spliced leader acceptor site (PAS or SLAS), weighted by the frequency of use of that site, using the PASs and SLASs sites and frequency we previously determined for analysis of ESB1[8]. PAS and SLAS usages are reported as site usage-weighted mapped reads spanning a site, normalised per million mapped reads (RPM)[8].

Inactive and active BES *ESAGs* and *VSGs* and MESs were identified from *T. brucei* Lister 427 2018 genome annotation[19] having confirmed BES1 is active. For plotting, the all *VSG* transcript set were defined by BLASTn search using each VSGnome[20] as query sequences, accepting hits at least 500 nucleotides long, at least 50% of the query sequence, and at least 50% sequence identity with the query. Distance to a BES promoter was taken as the distance to the closest upstream promoter.

Key resources: All plasmids, cell lines, antibodies, primers, and key reagents used in this study are listed in **Dataset S4**.

SI References

1. Savage, A.F., Kolev, N.G., Franklin, J.B., Vigneron, A., Aksoy, S., and Tschudi, C. (2016). Transcriptome profiling of Trypanosoma brucei development in the tsetse fly vector Glossina morsitans. PLoS one *11*, e0168877.

2. Christiano, R., Kolev, N.G., Shi, H., Ullu, E., Walther, T.C., and Tschudi, C. (2017). The proteome and transcriptome of the infectious metacyclic form of Trypanosoma brucei define quiescent cells primed for mammalian invasion. Molecular microbiology *106*, 74–92.

3. Naguleswaran, A., Fernandes, P., Bevkal, S., Rehmann, R., Nicholson, P., and Roditi, I. (2021). Developmental changes and metabolic reprogramming during establishment of infection and progression of Trypanosoma brucei brucei through its insect host. PLoS neglected tropical diseases *15*, e0009504.

4. Doleželová, E., Kunzová, M., Dejung, M., Levin, M., Panicucci, B., Regnault, C., Janzen, C.J., Barrett, M.P., Butter, F., and Zíková, A. (2020). Cell-based and multi-omics profiling reveals dynamic metabolic repurposing of mitochondria to drive developmental progression of Trypanosoma brucei. PLoS Biology *18*, e3000741.

5. Vasquez, J.-J., Hon, C.-C., Vanselow, J.T., Schlosser, A., and Siegel, T.N. (2014). Comparative ribosome profiling reveals extensive translational complexity in different Trypanosoma brucei life cycle stages. Nucleic acids research *42*, 3623–3637.

6. Jensen, B.C., Sivam, D., Kifer, C.T., Myler, P.J., and Parsons, M. (2009). Widespread variation in transcript abundance within and across developmental stages of Trypanosoma brucei. BMC genomics *10*, 482.

7. Faria, J., Glover, L., Hutchinson, S., Boehm, C., Field, M.C., and Horn, D. (2019). Monoallelic expression and epigenetic inheritance sustained by a Trypanosoma brucei variant surface glycoprotein exclusion complex. Nature communications *10*, 3023.

8. López-Escobar, L., Hänisch, B., Halliday, C., Ishii, M., Akiyoshi, B., Dean, S., Sunter, J.D., Wheeler, R.J., and Gull, K. (2022). Stage-specific transcription activator ESB1 regulates monoallelic antigen expression in Trypanosoma brucei. Nature microbiology *7*, 1280–1290.

9. Alsford, S., Kawahara, T., Glover, L., and Horn, D. (2005). Tagging a T. brucei RRNA locus improves stable transfection efficiency and circumvents inducible expression position effects. Molecular and biochemical parasitology *144*, 142–148.

10. Poon, S., Peacock, L., Gibson, W., Gull, K., and Kelly, S. (2012). A modular and optimized single marker system for generating Trypanosoma brucei cell lines expressing T7 RNA polymerase and the tetracycline repressor. Open biology *2*, 110037.

11. Alves, A.A., Gabriel, H.B., Bezerra, M.J., de Souza, W., Vaughan, S., Cunha-e-Silva, N.L., and Sunter, J.D. (2020). Control of assembly of extra-axonemal structures: the paraflagellar rod of trypanosomes. Journal of cell science *133*, jcs242271.

12. Burkard, G., Fragoso, C.M., and Roditi, I. (2007). Highly efficient stable transformation of bloodstream forms of Trypanosoma brucei.

13. Stanne, T.M., Kushwaha, M., Wand, M., Taylor, J.E., and Rudenko, G. (2011). TbISWI regulates multiple polymerase I (Pol I)-transcribed loci and is present at Pol II transcription boundaries in Trypanosoma brucei. Eukaryotic Cell *10*, 964–976.

14. Budzak, J., Jones, R., Tschudi, C., Kolev, N.G., and Rudenko, G. (2022). An assembly of nuclear bodies associates with the active VSG expression site in African trypanosomes. Nature communications *13*, 101.

15. Lansink, L.I., Walther, L., Longmore, S., Jones, M., Dowle, A., and Faria, J.R. (2025). Specialised RNA decay fine-tunes monogenic antigen expression in African trypanosomes. bioRxiv, 2025.2005. 2024.654301.

16. Hsiao, Y., Zhang, H., Li, G.X., Deng, Y., Yu, F., Valipour Kahrood, H., Steele, J.R., Schittenhelm, R.B., and Nesvizhskii, A.I. (2024). Analysis and visualization of quantitative proteomics data using FragPipe-Analyst. Journal of Proteome Research *23*, 4303–4315.

17. Alsford, S., and Horn, D. (2008). Single-locus targeting constructs for reliable regulated RNAi and transgene expression in Trypanosoma brucei. Molecular and biochemical parasitology *161*, 76–79.

18. Amos, B., Aurrecoechea, C., Barba, M., Barreto, A., Basenko, E.Y., Belnap, R., Blevins, A.S., Böhme, U., Brestelli, J., and Brunk, B.P. (2022). VEuPathDB: the eukaryotic pathogen, vector and host bioinformatics resource center. Nucleic acids research *50*, D898–D911.

19. Müller, L.S., Cosentino, R.O., Förstner, K.U., Guizetti, J., Wedel, C., Kaplan, N., Janzen, C.J., Arampatzi, P., Vogel, J., and Steinbiss, S. (2018). Genome organization and DNA accessibility control antigenic variation in trypanosomes. Nature *563*, 121–125.

20. Cross, G.A., Kim, H.-S., and Wickstead, B. (2014). Capturing the variant surface glycoprotein repertoire (the VSGnome) of Trypanosoma brucei Lister 427. Molecular and biochemical parasitology *195*, 59–73.
